# Leveraging Blockchain Technology for Ensuring Security and Privacy Aspects in Internet of Things: A Systematic Literature Review

**DOI:** 10.3390/s23020788

**Published:** 2023-01-10

**Authors:** Haider Dhia Zubaydi, Pál Varga, Sándor Molnár

**Affiliations:** Department of Telecommunications and Media Informatics, Faculty of Electrical Engineering and Informatics, Budapest University of Technology and Economics, Műegyetem rkp. 3., H-1111 Budapest, Hungary

**Keywords:** blockchain technology, Internet of Things (IoT), security, privacy, systematic, survey

## Abstract

As the Internet of Things (IoT) concept materialized worldwide in complex ecosystems, the related data security and privacy issues became apparent. While the system elements and their communication paths could be protected individually, generic, ecosystem-wide approaches were sought after as well. On a parallel timeline to IoT, the concept of distributed ledgers and blockchains came into the technological limelight. Blockchains offer many advantageous features in relation to enhanced security, anonymity, increased capacity, and peer-to-peer capabilities. Although blockchain technology can provide IoT with effective and efficient solutions, there are many challenges related to various aspects of integrating these technologies. While security, anonymity/data privacy, and smart contract-related features are apparently advantageous for blockchain technologies (BCT), there are challenges in relation to storage capacity/scalability, resource utilization, transaction rate scalability, predictability, and legal issues. This paper provides a systematic review on state-of-the-art approaches of BCT and IoT integration, specifically in order to solve certain security- and privacy-related issues. The paper first provides a brief overview of BCT and IoT’s basic principles, including their architecture, protocols and consensus algorithms, characteristics, and the challenges of integrating them. Afterwards, it describes the survey methodology, including the search strategy, eligibility criteria, selection results, and characteristics of the included articles. Later, we highlight the findings of this study which illustrates different works that addressed the integration of blockchain technology and IoT to tackle various aspects of privacy and security, which are followed by a categorization of applications that have been investigated with different characteristics, such as their primary information, objective, development level, target application, type of blockchain and platform, consensus algorithm, evaluation environment and metrics, future works or open issues (if any), and further notes for consideration. Furthermore, a detailed discussion of all articles is included from an architectural and operational perspective. Finally, we cover major gaps and future considerations that can be taken into account when integrating blockchain technology with IoT.

## 1. Introduction

The Internet of Things (IoT) domain includes a set of rapidly emerging communication, data processing, and insight generation technologies. It involves sensors and actuators of the physical world together with their communication means—sometimes under resource-constrained or environmentally harsh conditions. Furthermore, it involves data preprocessing and aggregation methods both at the network edge and in the cloud. Regarding human-centered, application-specific needs, the overall domain of IoT also includes methods for predictions, classifications, decision making, insight generation, and many more. Eventually, control processes are triggered based on these decisions, which initiate changes in the physical world, completing the working cycle of Cyber-Physical Systems (CPS).

IoT integrates appliances, services, sensors, actuators, etc., to offer connectivity solutions [1]. It also helps to improve the system’s efficiency by processing the collected data in real time [2]. However, it introduced many issues due to its resource constraints of connected devices and decentralized architecture [3]. IoT covers various application areas that revolve around people’s lives, such as the environment, healthcare, agriculture, transportation, and smart home, by revolutionizing surrounding objects to improve humans’ lives [4].

IoT requires solutions in many aspects in order to be considered secure, for example, physical security design, key management, client privacy, secure bootstrapping and transmission of data, authentication, and access control mechanisms [5,6,7]. Many approaches have been proposed to overcome the previously mentioned issues, such as a centralized server–client paradigm that relies on cloud servers. However, security and privacy aspects are still missing some pieces, and such features can be provided by blockchain technology.

We have reached a point for engineering systems where we need to answer both the traditional requirements for system security and safety [8] and the newly arising need for dynamic reorganization capabilities of supply chains and their system of systems [9]. It became inevitable to present a tenable solution that addresses the above-mentioned issues in IoT architecture to guarantee secure data exchange among IoT objects which requires trustless authentication, security, and robustness. Blockchain technology is one of the most trending approaches nowadays; it presents solid and robust features that can be utilized to overcome many limitations in different domains [10]. IoT ecosystem transactions can be managed securely using blockchain technology by eliminating the centralized entity by deploying distributed and public ledgers to allow anonymity in business models [11]. Blockchain enables data integrity and transaction transparency through a decentralized Peer-to-Peer (P2P) model. Many industrial and research domains are expanding their work on top of blockchain technology which results in higher efficiency compared to the traditional manner. An in-depth discussion is described in further sections.

Many papers have discussed the concept of blockchain technology and IoT, including systematic reviews, applications, challenges, and solutions such as [12] in general, and furthermore, in [13,14,15,16,17,18]. Moreover, further research addresses the integration of blockchain technology and IoT, which is also heading for advanced directions, including industrial and 5G support [19,20,21,22,23,24,25,26]. This paper will mainly focus on blockchain technology and IoT in a systematic manner to identify new perspectives and serve as a repository for the accumulated knowledge of these technologies in terms of research motivation, issues and challenges, solved gaps, the performance of these technologies in transactions and end devices, answering an important research question to identify the importance of using blockchain technology to boost the performance of IoT, and the usage of hybrid blockchains. Finally, the systematic manner included the recent and up-to-date approaches to identify the research applications and areas that have been focused on by the selected studies.

The rest of this paper is organized as follows: Section 2 introduces an overview of IoT and blockchain technology in terms of their architecture, network components, characteristics, and further features for each technology. Section 3 describes the manner used to include the research papers discussed in this review, including the search strategy, eligibility criteria, selection results, and characteristics of the included articles. Section 4 highlights the findings of this study which illustrate different works that addressed the integration of blockchain technology and IoT to tackle various aspects of privacy and security, and these are followed by a categorization of applications that have been investigated with different characteristics such as their primary information, objective, development level, target application, type of blockchain and platform, consensus algorithm, evaluation environment and metrics, future works or open issues (if any), and further notes for consideration. Section 4 also includes a detailed discussion of all articles from an architectural and operational perspective (Section 4.1, Section 4.2, Section 4.3, Section 4.4, Section 4.5, Section 4.6 and Section 4.7). Furthermore, in Section 5, we summarized the main lessons learned, covered major gaps, and shared future considerations that can be taken into account when integrating blockchain technology with IoT. Finally, the conclusion is presented in Section 6.

## 2. Overview

This section provides an overview of IoT and blockchain technologies, including their architectural design, protocols, consensus algorithms, characteristics, blockchain types, and IoT security and privacy concerns.

### 2.1. IoT

#### 2.1.1. Architecture

IoT is a combination of interconnected embedded sensors and heterogeneous devices, where they share common features such as limited processing capabilities, small memory, low power, and unique identifiers. IoT users can remotely provision data and access services through deployed gateways that connect the IoT network with the outside world [2].

**Figure 1:**
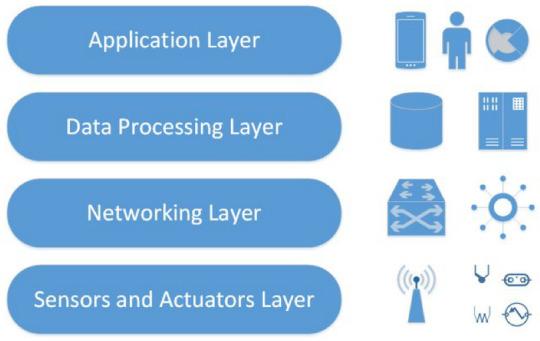
The generic architecture of IoT systems in a four-layered approach [31].

As stated by [27], “The Internet of Things allows people and things to be connected anytime, anyplace, with anything and anyone, ideally using any path/network and any service”. Various IoT architectures are proposed, each representing distinct perspectives and functions. From a deep technical perspective in wireless networks, ref. [28] described IoT architecture in a three-layer/tier manner, such as interfaces/services, network/ communication, and perception/hardware [29]. However, some other applied researchers and industrial experts consider a fourth layer. In [30], this is called the support layer, which participates in fog computing, smart computing, cloud computing, etc. Another interpretation of the fourth layer approach is depicted by Figure 1, where each layer represents different technology approaches and the scale of architectural elements [31].

From a top–down perspective, the four distinguishable key parts of generic IoT architectures are application, data processing, network, as well as sensors and actuators layers. To accomplish various applications (for example, healthcare, smart home, and smart transportation) of IoT devices, the application layer implements and delivers the results of the data processing (i.e., transport) layer [32]. The application layer is a user-centric layer that performs different functions on behalf of the user. The data processing layer analyzes the data acquired in the sensing layer and determines based on the findings. The data processing layer in various IoT devices (e.g., smartwatches, smart home hubs, etc.) also stores the results of earlier analyses to offer a better user experience. The network layer exchanges the results of data processing with other linked devices. The network layer facilitates sending data from the sensing and actuators layer to other connected devices as it serves as a communication channel. Data can be transferred across connected IoT devices using various communication technologies, such as Z-Wave, cellular network, Bluetooth, Wi-Fi, and Zigbee [33]. The primary function of the sensors and actuators layer is to identify any events occurring in the device’s periphery and to collect real-time data [32].

#### 2.1.2. Characteristics

The Internet of Things has various advantages because of its unique characteristics, such as the interconnectivity of heterogeneous systems, enormous scale, safety (e.g., healthcare and industrial domains), connectivity, dynamic changes, and things-related services. Heterogeneity refers to the use of diverse devices in IoT networks and hardware platforms; these devices are able to communicate with each other on various networks. Inter-connectivity refers to the ability to connect everything via global information and communication infrastructure. Safety refers to the systems affecting their external environment, including the physical well-being of individuals and the protection of personal data and endpoints. The enormous scale implies that the number of endpoints connected to each other through intranets and the Internet has risen significantly, which is majorly due to IoT devices. This growth requires further improvements in efficient data handling, clarified semantics, and data interpretation within applications. Network accessibility and compatibility are made possible via connectivity. Compatibility includes the control of protocol matching and data production and consumption interfaces. Accessibility means being able to reach the information anytime, anywhere, if authorization is provided and the stakeholder has authenticity. When a device is asleep or waking up, connected or disconnected, or in a specific place or at a specific speed, the state of the device changes dynamically, and the number of devices varies dynamically: this is what is meant by dynamic changes. Finally, things-related services include semantic coherence and privacy protection within device restrictions or constraints, which can be completed by changing the physical and information worlds’ technologies [34].

#### 2.1.3. Challenges

Although IoT has numerous benefits, it introduced many challenges that must be addressed, such as interoperability, scalability, heterogeneity, security, and privacy [35]. Many researchers have proposed various measures to enhance interoperability [36,37,38,39,40]. Interoperability describes the capacity of a system component’s technical requirements to work together effectively, regardless of how different they are. Scalability is introduced due to the fact that IoT is facing a tremendous issue in dealing with the rapid growth in the number of devices. It describes the system’s ability to handle future growth without negatively impacting its performance. Hence, when more devices are connected, scalability must be examined to see how the system can handle it. Refs. [41,42,43] are examples of studies on the scalability issue. Since the IoT network consists of a huge number of devices, it is a prominent illustration of the heterogeneity issue. When it comes to IoT, the primary goal is to provide a standard abstraction approach and maximize the functionality of connected devices. Due to the rapid expansion of IoT, there is a wide range of hardware and software configurations that the developers are striving to create an application that can work on top of them. Some examples of prior work to address the issue of heterogeneity are provided in [44].

New security vulnerabilities of system-of-systems appear due to the growth of IoT, which are caused by heterogeneity, decentralization, and individual vulnerabilities of IoT systems [45,46]. The complexity of deploying security mechanisms in resource-constrained IoT networks [47] resulted in difficulty in implementing traditional security techniques such as encryption, authentication, and authorization which might not be appropriate anymore. Furthermore, a complex cyber-physical system-of-systems may require autonomous approaches to handle security and safety issues [48,49]. Additionally, IoT devices are susceptible to malware activity due to the inability of security firmware to be updated on a timely basis [50]. In addition to security, it is difficult to maintain data privacy. There is a growing tendency to combine IoT with cloud computing, which provides IoT with additional storage power and computing abilities. However, data may be compromised if uploaded to third-party cloud servers, which are prone to privacy breaches [51].

Security and privacy aspects are the main focus of this paper because IoT has many issues within this area, and the research on integrating blockchain technology with IoT is mainly conducted to enhance these solution aspects. Managing security and privacy risks should be a top goal for increasing consumer acceptance of IoT applications. In addition, as IoT devices and related apps grow increasingly common in people’s daily lives, they must be completely secure. Security and privacy aspects in IoT may raise serious concerns due to a lack of proper authentication and authorization procedures. IoT protocols operate at different layers, which are a favorite target for hackers who always strive to identify new methods to intercept IoT connections even when proper authentication tools are used [28]. For example, possible attacks on each protocol in a specific layer include slowloris, cross-site scripting, HTTP flooding, DDoS, and repudiation attacks that target the application layer. The data processing layer is targeted with exhaustion attacks and targeted malware. The networking layer is further vulnerable to injection, smurf, SYN flooding, opt-ack, Sybil, sinkhole, wormhole, and other attacks. Further resource consumption, byzantine, and IP address spoofing attacks are in sight regarding the actual blockchain network. Finally, there physical damage or destruction, access control, and the disconnection of physical links are attacks toward the sensors and actuators layer [52]. Some attacks mainly target IoT layers based on the system, such as Wireless Sensor Networks (WSN) and Radio Frequency Identification (RFID). For example:

- When using WSN:
  – Physical/Link layer: Synchronization, selective forwarding, replay attacks.
  – Network/Transport layer: Sinkhole, false routing, eavesdropping attacks.
  – Application layer: Buffer overflow and injection attacks.
- When using RFID:
  – Physical/Link layer: Replay, sybil, passive interference attacks.
  – Network/Transport layer: Eavesdropping, impersonation, spoofing attacks.
  – Application layer: Tag modification, buffer overflow, injection attacks.

### 2.2. Blockchain

#### 2.2.1. Architecture

Blockchain is defined as a “set of chronologically ordered blocks” or a digital distributed ledger that maintains time-stamped transactions which are managed using unique algorithms to keep track of all blocks on the chain [53]. Each computer in the network is represented as a node where they share a duplicate copy of the data (“digital ledger”). All nodes in the blockchain utilize the same algorithm to reach an agreement called “consensus”. Blockchain technology operates in a distributed Peer-to-Peer (P2P) manner, which offers many advantages over traditional or centralized architectures, such as eliminating a single point of failure, which provides the network with high reliability and allows network nodes to work in a coupled manner which increases the computing power.

**Figure 2:**
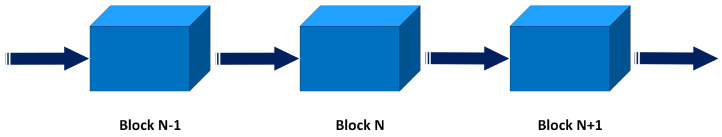
Blockchain structure.

Successive blocks of all transactions on the blockchain are linked together as depicted in Figure 2, where the previous block (N−1) is linked with the current block (N), which is also in turn linked to the next block that will be added to the blockchain (N+1). Additionally, blockchain technology has enabled the implementation of the “smart contracts” concept. It can be defined as computer programs or protocols that allow an agreement to be automatically enforced based on a set of specified conditions. The smart contracts specify the implemented application logic, making it an ideal component for extending blockchain technology to new domains [54]. Great examples of widely spread implementation for blockchains are Ethereum [55] and Hyperledger [56], which also include the capability of smart contract handling. In general, integrated blockchain technologies are designed to provide the following characteristics: decentralization, anonymity, autonomy, transparency, privacy, security, and collective verification [57].

#### 2.2.2. Consensus Algorithms

Consensus algorithms are essential to specify a set of rules and perform procedures when there is no mutual trust between network participants. By their very nature, they incentivize participating nodes to be trustworthy and produce or add new blocks to the blockchain. The use of consensus algorithm started and was utilized in cryptocurrency-based systems. Then, it was further extended to incorporate various applications, since each domain has its own set of requirements. Consensus algorithms represent the key function, demonstrating the methodology required to achieve absolute agreement between participants when verifying a new block. There has been increasing interest in existing consensus and replication processes, which can be used in blockchain systems [58].

Currently, consensus algorithms are employed in a variety of applications, such as banking and finance, supply chain management, healthcare, real estate, media, record management, and cybersecurity. Examples of consensus algorithms include: Proof of Work (PoW) [59], Proof of Stake (PoS) [60], Delegated Proof of Stake (DPoS) [61,62], Transactions as Proof of Stake (TaPoS) [60], Proof of Activity [63], Proof of Capacity, Byzantine Fault Tolerance (BFT), Replication [64], Practical Byzantine Fault Tolerance (PBFT) [65,66], Delegated BFT (DBFT), BFTRaft [67], Proof of Authority (PoA), Proof-of-Stake-Velocity (PoSV) [68], Proof of Burn [69], Proof-of-Personhood (PoP) [70], Proof of Bandwidth (PoB) [71], Proof of Elapsed Time (PoET) [72], Stellar Consensus Protocol (SCP) [73], Bitcoin-NG [74], Sieve [75], Ripple [76], and Tendermint [77]. Further details on different consensus algorithms, characteristics, advantages, and disadvantages can be found in [78,79,80].

#### 2.2.3. Types of Blockchains

Blockchain technology has been employed in a variety of applications and areas. From the access point of view, there are three types of blockchains, each of which serves a distinct purpose for specific applications: public, private, and federated.

Public blockchains have no centralized government or regulatory entities. The public chain has a high number of participating nodes, and its nodes’ trust level is the lowest of the three blockchain classifications. The public blockchain is employed in various IoT applications, including smart agriculture, smart healthcare, smart traffic, etc. A public blockchain is described as scalable, dynamic, and decentralized, and it supports over 100,000 nodes. However, it has many drawbacks, such as high latency, low throughput, high electricity consumption, and high computing power consumption, and it is susceptible to 51% of attacks [81].

Private blockchain: The private organization determines this type, and network nodes have varied permissions. The private blockchain is entirely controlled by a single entity, which has the authority to select the final consensus [82]. A private blockchain can reach consensus quickly and is able to resolve byzantine failures, but its complexity is high even if the number of nodes is low.

Consortium blockchain (federated blockchains): Participation, read, and write permissions are all governed by a set of rules. The consortium blockchain has fewer nodes than the public chain, but there is some trust among the nodes. This type is mainly used in the financial (banking) industry [83] and is gaining momentum in production-oriented supply chains [84]. These are becoming connected with Central Bank Digital Currencies (CBDC) in order to facilitate flexible digital payments for industrial partners [85]. Consortium blockchain solves the Byzantine failure problems and contains multiple consistency algorithms. The main disadvantage of this type is its high complexity.

### 2.3. Challenges of Integrating Blockchain Technology and IoT

Although blockchain technology can provide IoT with effective and efficient solutions, there are many challenges related to various aspects of integrating these technologies together. These include integration challenges with security-related system components, anonymity and data privacy, smart contracts, storage capacity and scalability, resource utilization, predictability, and legal issues.

Predictability is crucial in IoT because devices must be able to communicate with their surroundings in real time, which implies that the amount of time it takes for things to interact and the amount of latency between devices must be limited. Many consensus algorithms, such as PoW and PoS, are probabilistic when finalizing a transaction in the blockchain. At the same time, the confirmation confidence of the transaction in confusion is also probabilistic. Including predictability concerns in the blockchain, the design remains a key challenge. Predictability is essential for IoT-based healthcare applications [86]. For both manufacturers and service providers, the blockchain presents a severe issue because it connects individuals from diverse locations without any legal or compliance code to follow. Problems arise when private keys are retrieved or reset and transactions are reversed because there is a lack of rules on how to behave in such situations. It is unclear whether a worldwide, unique blockchain for IoT devices is intended to be governed by manufacturers or open to users in some IoT applications. Legal regulations are a crucial part when integrating blockchain with any other technology [87].

High heterogeneity and the lack of performance of IoT devices caused security issues at multiple levels. In addition, wireless communication and mobility are an additional set of properties that require security. More secure IoT design is even more essential due to the severe consequences caused by the growing number of attacks on IoT networks. Blockchain is widely viewed as a crucial technology for improving the security of IoT. However, the major barrier in integrating blockchain with IoT is the reliability of IoT devices’ data, because when the content of data is changed or damaged before it arrives on the blockchain, it will be stored as it is in the chain. Thus, blockchain is unable to identify and verify the integrity of data. Although data can be corrupted by malicious activities, it can be calculated in a wrong manner due to a failure in the devices themselves or any parts of them. Thus, before integrating IoT devices into blockchain technology, they must be properly tested to ensure that they will not cause damage to the system and must be placed in the correct location to prevent physical damage from occurring.

Anonymity and data privacy are critical issues for many IoT applications, especially when the device is related to a person, such as in e-health applications. In such applications, anonymity needs to be guaranteed, which is why blockchain is considered an ideal approach. Because data are collected and progress to application and communication levels, it is challenging to deal with data privacy even when many solutions have already been considered. Secure data storage is challenging due to the need for cryptographic software to be integrated into the device, which necessitates careful planning. They must take into account the devices’ limited resources and constraints on economic viability when making such enhancements. Because of the restrictions of IoT devices, many security protocols must typically be implemented using less limited devices, such as gateways. In order to accelerate cryptographic operations and reduce the burden on complicated secure software protocols, hardware cryptographic components could be used.

There is a corresponding increase in the size of the blockchain as there are more and more linked IoT devices that can generate massive amounts of real-time data, which results in a higher number of transactions and processes required to validate them. This issue might raise concerns in blockchain, since certain blockchain implementations can handle limited transactions per second. According to academics, deleting outdated transaction records from the blockchain’s storage can help solve the problem of scalability. In addition, researchers are attempting to redesign blockchain approaches in accordance with IoT constraints; for example, creating micro-blocks to store transactions and key blocks for leader election instead of the common block results in fierce competition among miners to control the micro-block generation process.

Although smart contracts are considered the next big thing in blockchain technology, several issues still need to be addressed. Although smart contracts may be useful in the IoT, their implementation in IoT applications varies widely because they are stored in a particular blockchain address as data and code. A transaction broadcasted in the network is required to alter the contract’s current state and hence the blockchain. Transactions must be signed by the sender and approved by the network before they can be added to the chain. The IoT could benefit from a secure and reliable processing engine provided by smart contracts. Using smart contracts results in secure and reliable processing. The logic of IoT applications may be securely modeled using smart contracts, but still, a few concerns must be addressed in the integration process. IoT’s constraints and heterogeneity must also be considered when implementing smart contracts. Furthermore, working with smart contracts necessitates relying on the oracles that offer real-world data in a trustworthy manner. IoT is unstable, making it difficult to validate these smart contracts. The use of many data sources may cause these agreements to become overburdened. Smart contracts do not share resources to deal with massive amounts of computing and distribution tasks, even when they are now characterized as decentralized and distributed. Smart contracts are executed on a single node, while code execution is performed on several nodes simultaneously. Instead of distributing tasks, this distribution is just used for validation.

In centralized designs, the consensus is guaranteed by a trusted authority, while in decentralized systems, a consensus is reached through voting and thus requires a lot of resources. The properties of IoT devices include low-bandwidth wireless connectivity, low power consumption, and low computing capabilities. Restricted resources should be allocated to establish an agreement in IoT instances where computationally intensive consensus procedures are unsuitable. A decentralized architecture can lower the total cost of the IoT system as opposed to centralized systems. There is a new resource wastage problem with blockchain, making integrating with IoT difficult. Consensus protocols in blockchain affect the number of resources needed. In most cases, these responsibilities are delegated to unconstrained devices that can deliver such capabilities, while other solutions assign such responsibilities to gateways. Alternatively, off-chain technologies could provide the functionality of transporting data outside the blockchain to alleviate the high latency.

Finally, it is crucial to mention blockchain trilemma (also called scalability trilemma), which indicates that scalability, security, and decentralization cannot be achieved concurrently in a public blockchain [88]. This issue is recognized since decentralization and scalability are inversely proportional in a blockchain with enormous numbers of participants. However, security and scalability are proportional when decentralization is fixed. Hence, trade-offs must be stated, since it is impossible to develop a blockchain with all features simultaneously. For example, Bitcoin currently can only process seven transactions per second while being secure and decentralized. Furthermore, although Hyperledger Fabric blockchain offers high transnational throughput and security, it is centralized. Fast and decentralized blockchains suffer from vulnerability to attacks. Current research efforts aim to explore improving blockchain scalability in layer one by improving the consensus algorithms (e.g., Ethereum 2.0) and using a concept called sharding. In layer 2, researchers seek to use nested blockchains and state channels to address this issue. Although blockchain trilemma introduces serious challenges, it is still a dominant technology due to its ability to support the required features to design an efficient and effective IoT scheme.

## 3. Review Methodology

This systematic literature review follows the principles suggested by Kitchenham and Charters [89] to perform the SLR to address the targeted research issues and to assure the transparency and reliability of this study. Because of the wide variety of blockchain applications, compiling literature to obtain a comprehensive picture of its various characteristics that make it offer to protect the Internet of Things is difficult. Thus, we focused on specific databases because exploring these large databases is partly facilitated by the number of articles and conference proceedings that can be accessed within them. Our review focused on the following databases:

- IEEE Explore Digital Library;
- ScienceDirect;
- SpringerLink;
- ACM Digital Library;
- MDPI;
- Wiley/Hindawi.

### 3.1. Search Strategy

The primary studies were gathered by using keywords to search the databases. We obtained a wide range of results since we used generic search phrases. Between the AND and OR operators, the principal search word is inserted. We considered the following search terms based on population and intervention: ((“Blockchain” OR “Blockchain Technology” OR “BC”) AND (“ Internet of Things “ OR “ IoT”) AND (“Privacy” OR “Security” OR “Confidentiality” OR “Integrity” OR “Availability” OR “Scalability” OR “Authentication & Data Protection” OR “Authorization” OR “Access Control” OR “Identity Verification”)). Between 6 and 15 October 2021, we ran a search that included publications published from 2018 onwards. Filtering reduces the number of relevant results returned by running a search query over multiple databases. Inclusion and exclusion criteria, outlined in the next section, have been applied.

### 3.2. Study Eligibility Criteria

Blockchain technology is being applied to the Internet of Things to improve privacy and security, and this study aims to summarize and assess those applications and uses. As a result, only the following studies were eligible to satisfy the selection criteria: a blockchain-based approach or technique that primarily aims to improve the security and privacy of the Internet of Things. Aside from that, other restrictions were put in place regarding publishing formats and languages used in the studies. Only peer-reviewed publications, conference proceedings, reports, theses, and dissertations published in English between 2018 and 2022 were included. Reviews, conference abstracts, commentaries, archived proposals, and editorials were all excluded. Finally, to provide a proper review, any article providing security or privacy for IoT using other approaches combined with blockchain technology was also excluded, as this study aims to leverage the benefits of blockchain technology only.

### 3.3. Selection Results

**Figure 3:**
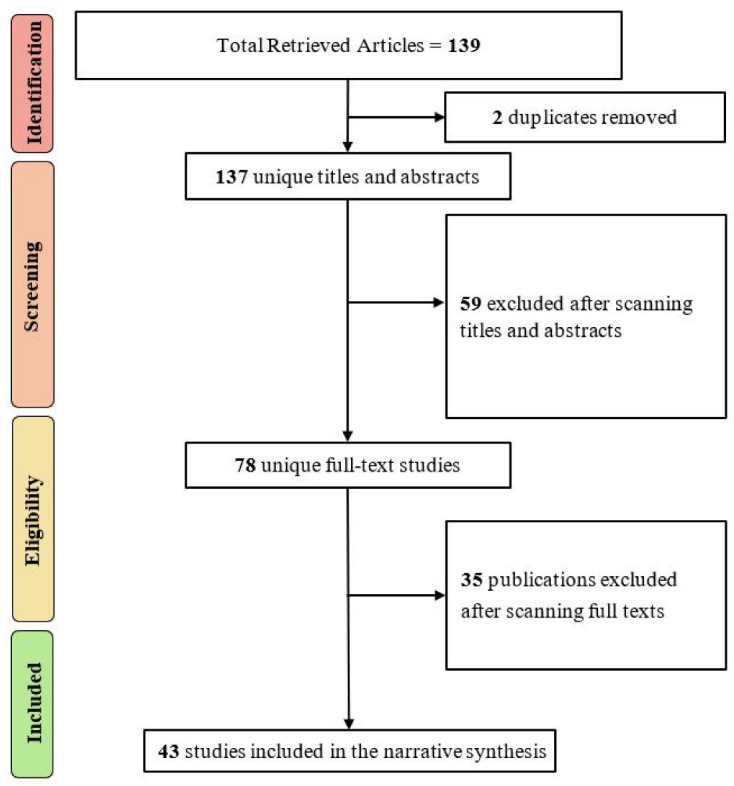
Study selection criteria.

There were two stages to the study selection procedure (screening title and abstracts of retrieved studies and screening full text of the studies selected in the first stage), as shown in Figure 3. We proceeded by screening the titles and abstracts of all the studies that had been obtained. After that, we read the entire collection of articles. First, we fully searched all studies that had been discovered in stage one. Consensus and discussion were used to address any disagreements among the reviewers. A total of 139 studies were found using our search keywords. Two publications with multiple versions were found, reducing the total number of articles to 137. The research pool is reduced from 137 to 78 articles when inclusion and exclusion criteria are applied to the title and abstract of each paper; thus, 59 articles were excluded. Finally, after scanning and reading full texts with inclusion/exclusion criteria for the remaining 78 publications, 35 publications were also excluded, bringing the total number of primary studies included in our SLR to 43 papers.

## 4. Findings

This section illustrates different works that addressed integrating blockchain technology and IoT to tackle various privacy and security aspects. We discuss the different characteristics of all included papers, such as their primary information, objective, development level, target application, type of blockchain and platform, consensus algorithm, evaluation environment and metrics, future works or open issues (if any), and finally further notes for consideration. We note that the calculations in our discussion depend only on our findings of the included papers.

**Figure 4:**
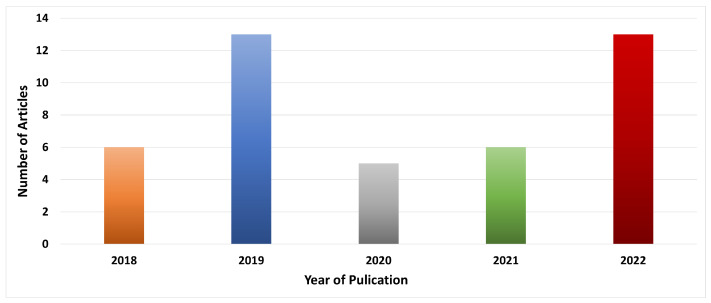
Distribution of the number of published articles by year of publication.

The primary information of the included papers [90,91,92,93,94,95,96,97,98,99,100,101,102,103,104,105,106,107,108,109,110,111,112,113,114,115,116,117,118,119,120,121,122,123,124,125,126,127,128,129,130,131,132] is shown in Table 1. Based on the data shown in Figure 4, we can observe that 13.95% of the total papers were published in 2018 [93,103,111,116,120,124], while 30.23% were published in 2019 [90,92,94,95,99,101,102,104,114,117,119,122,127], 11.64% were published in 2020 [91,96,113,118,121], 13.95% were published in 2021 [100,112,115,123,125,128], and finally, 30.23% were published in 2022 [97,98,105,106,107,108,109,110,126,129,130,131,132]. We note that blockchain technology research is considered new as it will require further development and testing in a real-time environment. In addition, the implementation of such systems is not easy. It requires more time than traditional architectures, which explains why blockchain-based approaches are not widely used in some research areas. However, many approaches that include blockchain technology in their designs are achieving promising results. Overall, 37.21% of the proposed schemes originate from China [92,94,95,96,97,102,104,105,113,114,119,124,125,128,129,132], and 62.79% are distributed between 14 countries [90,91,93,98,99,100,101,103,106,107,108,109,110,111,112,115,116,117,118,120,121,122,123,126,127,130,131]. Most of the included papers are published in journals, which result in 79.07% [90,91,94,96,97,98,99,100,102,104,105,106,107,108,109,110,111,112,113,114,115,118,119,121,122,123,125,126,127,128,129,130,131,132], while 16.28% are published in conferences [92,93,95,101,103,116,120], and 4.65% are published in book and symposiums [117,124]. We included a detailed description of the publishers (name of journal or conference) to address the common databases used. Overall, 51.16% are published in the IEEE database [91,92,96,97,100,101,102,104,105,106,107,108,110,111,116,119,120,123,124,127,128,132], 20.93% are published in Elsevier [94,103,112,113,114,117,118,121,122], 13.95% are published in MDPI [98,99,109,115,130,131], 6.98% are published in Springer [93,95,125], and 6.98% are published in Wiley/Hindawi [90,126,129].

**Figure 5:**
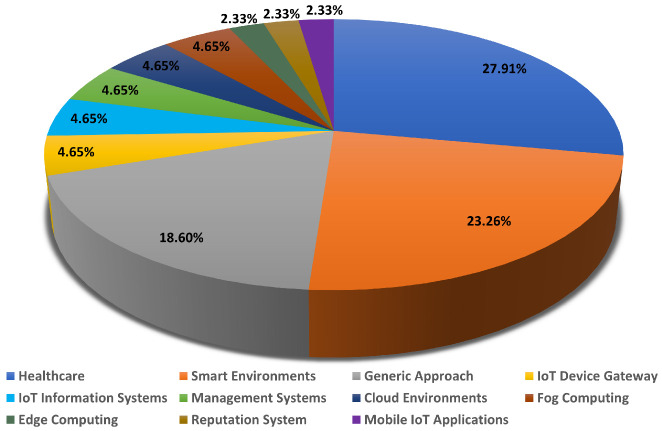
Article distribution of the included studies by target application.

The objective and development level are presented in Table 2. Based on the data shown in Figure 5, our results show that 27.9% of the included studies focused on the healthcare domain [99,100,101,102,103,104,105,106,107,108,109,110], while 18.6% focused on proposing generic approaches [91,92,93,94,95,96,97,98]. From the total percentage of the included articles, 23.25% aimed to target smart environments applications divided into 9.3% for smart home applications [120,121,122,123], 6.98% presented systems designed to target smart cities [116,117,118], and there were 2.32% of each of the following: smart factory [119], smart traveling [124], and smart agriculture [130]. Furthermore, the IoT device gateway [111,112], IoT information systems [113,114], management systems [131,132], cloud environment [128,129], and fog computing [125,126] have carried out 4.65% for each separate application. Finally, the rest (≈6.99%) aimed to address edge computing [90], mobile IoT applications [115], and reputation systems [127].

Regarding the development level, we used Technology Readiness Level (TRL) as the base concept to describe the development level of the included articles. TRL includes nine values where each value represents the technical maturity of a technology; every three values describes a particular research phase. TRL phases are defined as research, development, and deployment. In order to reduce the complexity and initiate an understating of measuring what level each system is developed, we determined an appropriate value for each phase. For the research phase, 2 is selected, which indicates that the concept has been formulated. For the development phase, 5 represents the validation process in a relevant environment. Finally, for the deployment phase, 7 refers to the demonstration of a prototype in an operational environment. Overall, 79.09% proposed implemented design models (evaluated using simulation tools) [91,92,94,95,96,97,98,100,102,103,104,105,106,107,108,109,110,111,112,113,114,115,118,120,121,122,123,125,126,127,128,130,131,132], 18.6% proposed a concept formulation or use cases with the further intention to implement and evaluate them [90,93,99,101,116,117,124,129], and only one approach was implemented in a real-time environment [119]. The included approaches aimed to achieve various goals to enhance certain aspects (security or privacy) of IoT. For example, ref. [90] investigated the delay concerns, [93] discussed access control using a firmware update approach, ref. [94] presented a Privacy-preserving Thin-client Authentication Scheme (PTAS) to address privacy issues, heterogeneity and scalability issues are also discussed in [96], whereas ref. [99] aimed to address the access control for EHRs, a multi-tier blockchain framework for privacy-preserving EHRs is presented in [103], a data integrity check approach that does not require trusted third parties is designed using blockchain, bilinear pairing, and the Lifted EC-ElGamal cryptosystem in [113]. Some approaches focused on introducing lightweight techniques such as [114,121,122].

The used blockchain platform, blockchain type, consensus algorithm/protocol, evaluation environment, and metrics are presented in Table 3, Table 4, Table 5, Table 6 and Table 7. Different platforms are used for each application: 37.21% of the included studies focused on using a generic platform or Ethereum [91,93,95,97,99,104,106,111,113,115,116,117,120,123,127,130], 20.93% were implemented using Hyperledger Fabric [93,98,105,108,109,110,118,125,128], while 23.25% focused on special or other types of blockchains [90,92,94,96,100,102,121,122,124,132]. Note that some studies allow or use more than one platform [93,102,110]. It indicates that generic platforms do not always fit the requirements of specific approaches; thus, other platforms are required to ensure that the proposed architecture achieves its optimal performance. Blockchain type is also based on the domain requirements and the design itself. Overall, 41.86% of the studies are using private blockchain [90,91,92,93,96,100,101,106,109,111,115,118,119,120,123,124,125,126], which indicates that it is more suitable for the desired applications since it offers more control and security for the network, while 25.58% are using a public blockchain [91,94,97,102,115,116,117,121,122,127,130]. In addition, 37.21% of the approaches allow the implementation using both types or a combination of both (consortium blockchain) [91,95,98,102,103,105,106,107,108,110,114,115,126,128,131,132]. From our perspective, private blockchain offers the most needed features that are required to reach a high level of security and privacy.

Although PoW is the most common consensus algorithm, we can observe that only 25.58% of the total approaches used it [94,96,102,108,115,117,119,120,123,127,130] since it has many disadvantages. We note that some studies used a modified version to offer a better solution, such as [96], or added another algorithm to support it, such as [96,102,108,115]. Note that modifying the common algorithms such as PBFT can result in an efficient model [114]. Some approaches used a time-based consensus algorithm to achieve higher security and privacy [121,122]. It can be observed that there is still a need to propose new consensus algorithms to address the drawbacks of IoT because the requirements for this technology are not similar compared to other technologies. Thus, it is vital to tackle this issue; for example, one of the included studies designed their consensus algorithm named “Three-Dimensional Greedy Heaviest-Observed Sub-Tree (3D-GHOST)” and implemented it with a modified PoW [96], Proof-of-Epidemiology-of-Interest (PoEoI) was used in [107], and other studies used Zero-Knowledge Proof (ZKP) [110], time-dependent consensus algorithm [121], Distributed Time-based Consensus algorithm (DTC) [122], and combined RBFT and Raft [129]. Furthermore, BFT, PBFT, and its modified versions are commonly used nowadays [90,98,101,102,109,114,126,128,132] due to the advantages offered; for example, PBFT reduces energy usage and eliminates the need for confirmation to perform transactions.

In the evaluation environment, we aimed to cover software tools and certain important hardware devices used in the implementation and experimentation phases. Different tools are used such as Solidity [91,97,110,127,131], Web3.js [91,120,128], various types of sensors [91,100,123,130], gateways [92,111], smart devices [94,102,115,126], Node.js [112], and NS3 [121,122]. C++ is used as a programming language in [92,121], Go and MATLAB are used in [98,100,125], Python is used in [96,102,109,113,115,123], and Java is used in [94,102,111,132]. For detailed information, refer to Table 3, Table 4, Table 5, Table 6 and Table 7 and the references listed for each environment. Regarding the metrics used to evaluate each study, time was used as one of the parameters in [92,97,100,103,104,105,106,107,110,111,113,114,115,118,121,122,128,131,132], while throughput is used in [96,98,108,109,110,114,118]. Many approaches evaluated their approach by testing it with more specific parameters such as AES encryption and decryption rate [95,112], validation of ACL rules [118], effectiveness and feasibility [102], computation cost for PDSS [111], functionality [94], probability of the illegality [113], latency [114,118], bandwidth consumption [115], IoT elements activity [100], bit error rate [125], cost [97,102,105,107,108,127,128], K-fold cross-validation [123], DTM in the overlay [122], sustainability [110], different latency metrics [130], and calculations based on credentials [132].

As mentioned earlier, we focused on the target application when implementing the taxonomy of this study. The included studies proposed different solutions to address IoT applications’ privacy or security aspects. In addition to the detailed information in the above tables and figures, in the following subsections, we will discuss each study from an architectural and operational perspective to ensure that this review provides a comprehensive overview.

### 4.1. Generic Approaches

Multiple studies focused on proposing generic solutions to address certain features within IoT. An Ethereum-based approach was proposed to address layer-wise security issues and device authentication in IoT applications [91]. This work focused on specific aspects, including eliminating the idea of localizing authorization and authentication inside the IoT network and eliminating latency issues on the IoT network. Blockchain operation is only required when adding a new user or device is needed. Blockchain is the only entity that allows creating permissions regarding the scaling of IoT networks.

In [92], the authors create a safe, fine-grained access control strategy for users, devices, and data. Then utilize smart contracts to implement the strategy. System design includes an access control strategy, user registration and authorization, device’s safe insert, database, smart contract design, and transaction design. The access control strategy consists of three tables defining the access rights: the user access table, device resource table, and role table. The device’s safe insert ensures that the device’s hardware includes embedded identity information. The database scheme stores data collected by devices and data related to the behavior of the user, device, and gateway. Three types of smart contracts are designed in this scheme: user access, device insert, and log. The design of transactions describes the details that must be encapsulated in the message to be sent.

Another approach focused on proposing a mechanism to address the access control management of how users deal with their data [93]. This model consists of the main blockchain network, off-chain storage, aggregators (publish), subscribers (subscribe), and vendors. Aggregators publish data and define how third-party can access the data. Third-party, known as subscribers, can access that data through transactions. Off-chain storage stores the data published by aggregators through a scheme called content-based addressing. Manufacturers of IoT devices are known as vendors, and they are responsible for distributing official firmware. In order to manage access permissions and update new firmware, the blockchain network is equipped with two smart contracts: FirmwareUpdate and AccessControl.

The PTAS scheme [94] is proposed to ensure privacy using private information retrieval and security using (m-1)-private PTAS to protect against a collision of network nodes. This scheme allows thin clients to function normally as full-node users by hiding user identity in k indistinguishable identities. Security and functional comparisons are conducted to highlight this scheme’s high level of security and comprehensive functionality compared to other schemes. However, PTAS improves safety while sacrificing little efficiency.

IoTChain [95] is a scheme proposed to protect the security of IoT information based on blockchain technology characteristics and the AES encryption algorithm. The large-scale secure storage of IoT information data can be provided by IoTChain, which can authenticate and grant access to authorized users. As a result, the researchers in this study proposed efficient and secure authentication, privacy protection, and multi-signature conditional traceability solutions based on blockchain technology.

Spacechain [96] is a blockchain architecture with a three-dimensional ledger that deals with the scalability and heterogeneity of IoT networks. They also proposed a consensus algorithm called 3D-GHOST to improve network performance and security. Macro-blocks are used to create Directed Acyclic Graph (DAG) to provide the system with the third dimension aspect. DAG consists of a vertex, edge, ack-edge, and ref-edge, which illustrates the operation of this foundation. In the data structure design, the validation process occurs in three steps: consensus algorithm validation and verification using PoW, header_hash validation, and timestamp validation. For the consensus algorithm, the blockchain is divided into the main-chain and side-chain to ensure better performance. A novel DWD mechanism is used for dynamic weight distribution with many metrics, such as Cardinal Value (CV), Data Validity (DV), and Contact Degree (CD). This architecture is implemented and evaluated; it results in a better performance than the NKC scheme.

A study proposed a blockchain-based privacy-preserving and trust-centric approach and Proof-of-Trust (PoT) consensus algorithm to tackle the challenges related to trustworthiness and create an affordable and lightweight consensus mechanism [97]. This study included a trust evaluation mechanism, PoT consensus algorithm, and privacy protection mechanism. The commitment scheme and ring signature combine to create a robust privacy protection mechanism. On the other hand, PoT is designed by connecting the trust value of network miners with mining difficulty. The design of the proposed DSA system included four phases: individual sensing, sensing fusion, spectrum allocation, and spectrum access. This system offers decentralization, transparency, automation, and flexibility. The proposed consensus algorithm increased the scalability and reduced computation cost.

With blockchain technology, a lightweight multi-chaincode model is proposed to address central authority management issues that lead to a lack of privacy, low scalability, and single point of failure [98]. The proposed system includes various layers, such as Consortium Blockchain Manager (CCBCM) for access control, an Aggregated Edge Blockchain Manager (AEBCM) layer for communication purposes, and Edge Blockchain Managers (EBCMs) that contain network devices. To achieve the required scalability, low latency, and high throughput, a hierarchical permissioned blockchain is used. EBCM is used within the cluster to manage the data securely. This model eliminates Trusting Third Parties (TTP) by incorporating self-executed smart contracts. The authors provided a security analysis discussion on how the proposed model offers availability, integrity, and confidentiality.

### 4.2. Healthcare

Dealing with big data in the healthcare domain can raise security and privacy issues, endangering the patient’s life. A novel privacy-preserving framework to secure the analysis and management of healthcare data is proposed [99]. This study addresses the IoT devices’ constraints and how to resolve the issues requiring extra computational power, high bandwidth, and computation cost. The proposed framework consists of healthcare wearable IoT devices, smart contracts, healthcare providers, cloud storage, and an overlay network. Asymmetric and ARX symmetric encryption schemes are both used. Signature correctness and signers’ anonymity are achieved using lightweight ring signature technology. Further work can be completed to implement this framework in a testable environment and provide more security guarantees.

BIoTHR [100] is an EHR management system based on private blockchain to ensure the timely monitoring of reliable and secure data transmission. This scheme supports full EHR utilization, a swarm exchange network for IoT-based implementation, UML activity modeling, and trusted parties. Five sensor nodes are employed to aggregate patient information to provide the network with heterogeneous features. The authors included a detailed discussion of a novel swarm exchange paradigm to create a tamper-proof and robust system. Different algorithms were used to create the private blockchain, swarm listening, address announcement of swarm local listening, address announcement of swarm interface listening, swarm connection opening, and connection closing. This study provides protection of data privacy, protection against fraud, security and transparency, interoperability, access control, pseudonymity, full decentralization, high availability, design simplification, and reduced cost. This design can be improved to function in a large-scale network, API is required, and a proper manner of mining is needed.

Another study that addresses access control is proposed using blockchain architecture for e-health applications [101]. The general blockchain structure was modified to make this approach fit the healthcare domain, reduce data redundancy by clustering network miners, and reduce transaction size to reduce network overhead; a pseudonym is assigned for each patient, and the data are stored in the nearest location to address security and privacy challenges. This model consists of sensors, Personal Digital Assistance (PDA) or a smartphone, the IoT Health Manager (IHM), a central server responsible for managing the data, healthcare institutions, a blockchain network, and miners. Further implementation and experiments are required to evaluate this model.

To eliminate the issue of large-scale networks, Healthchain [102] is a privacy-preserving scheme proposed for large-scale health data to achieve fine-grained access control. This scheme consists of IoT devices, user nodes, doctor nodes, accounting nodes, storage nodes, Userchain, and Docchain. This design aims to provide high efficiency, privacy preservation, accountability, and on-demand revocation. The blockchain network (referred to as Healthchain in this design) is divided into Userchain and Docchain (called subblockchains). Userchain is implemented to prevent tampering with users’ transactions; it contains IoT transactions and key transactions. Diagnostic transactions are the only ones supported by Docchain and are secured by the diagnostic key assigned to each user. Keys were decoupled from encrypted data to make key management more flexible. In order to protect users’ privacy, they can revoke doctors’ access to their records at any moment. This scheme provides privacy preserving, accountability, and revocability. This study meets the standard security criteria according to its security analysis. The performance evaluation results suggest that Healthchain is a feasible and efficient solution.

Pseudonym-Based Encryption with Different Authorities (PBE-DA) [103] is a novel protocol that allows patients to manage their EHR data securely and provide the perfect privacy preserving. The proposed architecture consists of three tiers, namely network nodes (constrained and unconstrained), number of authorities (medical institutions and organizations), and EHRs cloud providers (servers). PBE-DA is designed in a multi-tier blockchain framework that uses Elliptic Curve Cryptography (ECC). The fog or access layer is the initial tier to connect devices and patients using a gateway. The ledger distribution and communication of different EHRs are analyzed in the second tier. Finally, compliance issues between EHR providers are examined. MIRACL security tools are used to evaluate the framework for various security functions.

A study discussed how the volume of medical imaging is increasing, which might affect the diagnosis and treatment because these images must be retrieved first [104]. This study presents a threat and a layered architecture based on blockchain that selects feature vectors to handle large-size images. To ensure the privacy of medical images and their features, a customized transaction structure was designed in addition to the feature vector. This study focused on three types of threats from a security perspective: data forgery, data tampering, and privacy disclosure. The system design includes five entities: hospital, third party, image retrieval service, regulatory authority, and miner. Transaction generation, image feature encryption, and image feature extraction are the main components of the transaction layer. Based on the encrypted image characteristics, the service layer provides crucial functionality for similarity measurement and image retrieval.

Introducing the medical field to IoT has led to reduced cost, increased accuracy, and improved efficiency; security and privacy aspects are still essential concerns due to the heterogeneous network that contains various entities and a large amount of data. One possible solution is to introduce an IoMT authentication framework integrated with blockchain technology to create a general architecture that can eliminate the issues mentioned earlier [105]. Elliptic Curve Cryptography (ECC) and Physically Unclonable Functions (PUFs) are authentication schemes between system components. Five phases are included in the proposed schemes: revocation phase, password and biometrics update, login and authentication, registration, and system initialization. Multiple procedures are performed in these phases, such as creating a blockchain network, setting up the cryptographic parameters, registering the entities with the Register Center (RC), initializing authentication between entities, updating certain information, and summarizing the actions to be performed when a private key is lost or compromised. The proposed scheme achieves the desired security and operational requirements based on the security and performance analysis.

Regular and remote monitoring of patients with chronic diseases is critical due to their unpredictable health conditions. Metrics such as scalability, processing time, and security are essential when implementing a blockchain-based and proxy re-encryption healthcare system [106]. The proposed system architecture comprises hospitals, physicians, and patients linked with the ministry of health through the blockchain network. IPFS is used to store the collected and encrypted health data. Patients are supported with IoT medical devices that collect health data and a smartphone that acts as a bridge with the medical entities. To speed up the consensus process and data storage, the Clique PoA algorithm is implemented in the system. Compared to the state-of-the-art methods, the proposed system offers high security.

GarliMediChain [107] is a health data-sharing anonymous system that ensure privacy, anonymity, and low latency by integrating blockchain technology with garlic routing. In addition, to maximize institutions’ payoffs, a coalition system is introduced. Fictitious play is used to enforce trust among coalition groups. Furthermore, Proof-of-Epidemiology-of-Interest (PoEoI) is a new consensus algorithm proposed to select miners and generate blocks based on an addition number game. The proposed system consists of five components: the fictitious play, a learning paradigm, a coalition group, a consortium blockchain, garlic routing that hides the identities of communication entities, and edge nodes to connect smart devices. The simulation results demonstrate that the proposed system is robust against attacks and efficient.

To achieve distributed consistency in a peer-to-peer (P2P) environment, an architecture called BIoMT [108] is proposed, which consists of consortium blockchain built on top of Hyperledger Fabric to provide provenance, transparency, integrity, and security for serverless P2P. Distinct operational controls are implemented using different protocol types to reduce resource consumption costs. Moreover, a new lightweight consensus algorithm is proposed based on PoW; the proposed algorithm utilizes the predefined policies of Hyperledger Fabric to reduce the transmission bandwidth and the required computation power. The proposed system architecture contains a serverless network to manage network resources required to complete a process. The BIoMT node is responsible for managing the records until submitting them to the filecoin, representing immutable storage belonging to a third party. On-chain and off-chain designs are provided for the communication protocols. The Hyperledger Fabric expert handles real-time medical transactions. Two storage designs are included to eliminate any capacity issues, primary and secondary. The experimental results demonstrate that BIoMT reduced the resource constraints.

Hiding sensitive data from malicious parties requires advanced methods which can be utilized from Information Hiding Techniques (IHT). When combining IHT with smart contracts and blockchain technology to create a framework for the medical supply chain, security and privacy aspects are enhanced [109]. This study proposes a different method of encrypting the information into other auxiliary messages using improved steganography techniques. Multiple pre-authenticated healthcare providers are merged into a private cluster in the blockchain network, and only entities inside the network are allowed to communicate and participate in the processes. Using smart contracts, one-time secret keys are securely created and distributed among related parties. The proposed framework comprises cluster pre-selection, hash key registration, and smart contract phases. The proposed system architecture is divided into cloud, fog, edge, and healthcare IoT device layers. This approach ensures lower execution time with higher security than other classical approaches.

A model that combines Self-Sovereign Identity (SSI), Verifiable Credential (VC), Decentralized ID (DID), Attribute-Based Access Control (ABAC), Role-Based Access Control (RBAC), and blockchain technology called Decentralized Self-Management of data Access Control (DSMAC) [110] is proposed to allow patients to control their medical data. For emergency cases, advanced access control techniques are implemented using verifiable credentials and decentralized identifiers. In addition, role-based access control policies are conducted by leveraging smart contracts. A DID document is used to create an attribute-based access control mechanism. The proposed framework comprises three layers: the user layer, the F2C layer, and the IoMT devices layer. Based on performance evaluation, the proposed framework is efficient and scalable regarding cryptographic computations, latency, throughput, and execution time.

### 4.3. Smart Environments

#### 4.3.1. Smart Home

SHIB [120] is a smart home based on the IoT-Blockchain that addresses the challenges related to the ability of extension, trust access control, and data privacy. Only the creator of ACC can add new policies, update existing ones, or remove privacy policies from the ACC blockchain. In order to use the SHIB architecture, a smart homeowner must have agreed to a smart contract with the other parties involved. Using defined policies, smart contracts are able to restrict access requests when misbehavior is sensed in the network to increase the security and privacy of home data. Compared to other existing models, this design contains a Judge Contract (JC) that can perform judgment and impose penalties on misbehavior.

ELIB [121] is a model proposed to eliminate specific issues associated with blockchain technology, such as high bandwidth, limited scalability, and high computation complexity, and implement an efficient smart home design that fits IoT necessitates. Smart homes with limited resources benefit from a centralized manager that produces shared keys for data transmission and processes every incoming and outgoing request. An overlay network is generated as shown in the current ELIB model; high-equipped resources can merge with a public BC that guarantees devoted security and privacy. The suggested ELIB model includes three optimizations: a Distributed Throughput Management (DTM) strategy, certificateless cryptography, and a lightweight consensus algorithm. Based on the experiments with several parameters, ELIB demonstrated excellent performance.

LSB [122] refers to a “Lightweight Scalable Blockchain” that utilizes overlay networks to achieve decentralization and end-to-end security. Network nodes are grouped into clusters using a clustering algorithm (similar to [137]). A Cluster Head (CH) is elected in each cluster; it represents the node with maximum coverage (neighbors). CHs are called Overlay Block Managers (OBMs) because they manage the blockchain network. A genesis transaction must be created by overlay nodes using one of the following approaches: certificate authorities and Burn coin in Bitcoin. A genesis transaction is broadcasted from one OBM to another after verification. In order to reduce delay and mining processing overhead, they designed a consensus algorithm called distributed time-based. Cluster heads are responsible for efficiently employing the distributed trust approach among network nodes to verify new blocks. A distributed throughput management algorithm is used to ensure that the network throughput is stable enough (based on specific parameters). LSB is designed to fulfill IoT fundamental requirements such as connectivity and mobility and real-time applications. It is implemented in different scenarios that include high-resource devices and low-resource devices. The authors analyzed and discussed further aspects of LSB, such as OBM reward, auditability, and complexity. According to a security assessment, LSB is highly fault-tolerant and secure to a wide range of attacks. Further development is required to evaluate this model in real-world settings.

A privacy-preserving authentication scheme is proposed to illustrate how data are collected and shared in smart home applications [123]. The proposed scheme combines three base concepts to create a secure framework: edge computing, smart contracts, and attribute-based access control. Data are transferred to the cloud securely and privately using a differential privacy method which offloads systems’ heavy processing; eventually, the system scalability is increased. The proposed system architecture consists of end users, IoT devices, multi-edge servers, and the cloud. Two types of contracts are used in the attribute-based access control: register contract and access contract. The authors explained how transactions are being carried out; four phases are used: chain transaction, state delivery, request control, and initialization. The differential privacy enhancement mechanism includes a plain algorithm, private algorithm, dataset, and implementation. The proposed approach performs better than the existing scheme; it provides efficient security, privacy, resiliency against attacks, fine-grained access control, and less computing cost.

#### 4.3.2. Smart City

A use case is presented in [116] to address privacy exposure and security threats of cyberinfrastructure in a smart city. This study discusses IoT-based access control first using two primary models; Discretionary Access Control Models (DAC) and Mandatory Access Control Model (MAC). DAC explains how to transmit the rights of the object from one to another; MAC refers to classifying objects in the system and how to regulate access among them. This study compares the difference between implementing this model in traditional and blockchain-based architectures. The process of exchanging data starts between actors (user and organization or two users where one is outside the infrastructure), the data are transmitted to IoT cyberinfrastructure, which is followed by the private cloud and blockchain network. The user needs to encrypt only the part of the data that can be shared. This study is still in the early research phase and must be investigated further. A study on the smart city security model is illustrated in [117], covering the theoretical aspects. The authors started discussing data management and distribution, which were followed by communications, private key management, securing third parties, smart contracts (automation of procedures), and protocols.

A privacy-preserving innovative framework called PrivySharing [118] is proposed to secure IoT data in a smart city environment. Privacy is preserved as each channel has a finite number of approved organizations and processes a specific type of data (financial, health, energy, etc.). Private data collection and encryption are used to isolate further and secure data within a channel. A private data collection methodology is adopted to ensure the privacy of critical data by sending the data directly to the authorized requesting node (NMSP). A Membership Service Provider (MSP) defines the access rights and which RCAs/CAs are trusted. A different Ch is used for each data type to ensure decentralization, scalability, and privacy. This design provides the concept of “right to forget” regarding user data, efficiency in terms of computational requirements and energy consumption, user-defined fine-grained access control, allowing users control over their data while providing an auditable network operation, blockchain access through API, and reward system for data sharing.

#### 4.3.3. Smart Factory

A multi-center blockchain-based security and privacy model is proposed to reshape traditional IoT architecture for smart factories [119]. The proposed architecture consists of five layers: application layer, firmware layer, storage layer, management hub layer, and sensing layer. Users are provided with different services by the application layer. The firmware layer is used to connect all layers through underlying implementation technologies, data are stored in a distributed form in data centers represented by the storage layer, the process of managing the data and creating blocks is completed by the management hub layer, and finally, the process of obtaining data and preprocessing occurs in the sensing layer using sensors with microprocessor (computing power). This architecture is divided into intranet and extranet; the first deals with data collection and storage, and the latter aims to offer users different services by utilizing the data. This model is designed with a private blockchain where all nodes are trusted initially; thus, it does not include a reward mechanism or competition. The block structure is created with two parts: block body and header (stores structured data and its attributes). Finally, the authors combined two models: Biba and Bell-La Padula (BLP) to ensure CIA requirements.

#### 4.3.4. Smart Traveling

Due to the massive data generated and the vast scale of IoT networks, data fusing and privacy are still significant challenges. Thus, an inter-cloud data fusing and privacy-protected platform based on JointCloud is proposed to address the analytic activities and data mining of IoT [124]. The authors discussed two main platforms implemented on single clouds: Baidu and Amazon AWS. Then, they presented their design based on JointCloud Computing (JCC) because it is more suitable for constructing complex applications. This framework can be broken down into three tiers. The first tier is made up of a variety of sensors that are linked to several clouds. In the second tier, JointCloud Collaboration Environment (JCCE) links clouds together. Services are located in the third tier, based on the JCC, and each user is provided with an application and personalized service. This platform offers enhanced security because data are stored in a private cloud. In addition, it eliminates privacy disclosure and prejudice because, in JCCE, trades are automatically executed.

#### 4.3.5. Smart Agriculture

A blockchain-based application is implemented to store malicious information to prevent future attacks for a smart-farm security monitoring framework [130]. The application consists of three layers: the smart farm layer, the cloud layer, and the blockchain layer. The smart farm layer consists of different sensors to collect data, and the cloud layer is responsible for processing sensors’ events and retrieving the required information. The blockchain layer comprises the Ethereum blockchain with smart contracts to check environmental conditions and store farming data. Ethereum nodes perform the mining process; an entity or individual controls these nodes. The cloud layer consists of an AWS cloud, Anomaly Lambda Function, and Infura Ethereum API that runs smart contracts and connects the middle layer with blockchain layer nodes. This framework is implemented to work with only one consensus algorithm: Ethereum proof-of-work (POW). Based on performance evaluations, this prototype resulted in nominal network latency.

### 4.4. IoT Device Gateway

A blockchain-based connected gateway design is presented in [111] for BLE-based devices to address privacy preferences in IoT networks. Each user has to consent to data access by any third party using the gateway to prevent privacy leakage. Furthermore, a robust digital signature technique is presented to facilitate the secure management and authentication of privacy preferences. The proposed blockchain gateway consists of gateway administrators, end-users, and administrators or owners. The administrator stores the information of all devices in the network and their privacy policies: for example, device features, manufacturer information, unique device name, and other attributes. The architecture of the blockchain gateway consists of the user interface and administrator interface and the internal components that can be managed from these interfaces. In the proposed gateway design, device binding refers to the administrator’s registering or adding a new device. Later, a new Proposed Digital Signature Scheme (PDSS) is proposed based on robustness and intractability using bilinear pairing and ECDLP. PDSS is realized using six phases. Furthermore, this study discussed the privacy preference preserving concept and intelligent access control on IoT devices. Detailed evaluation scenarios are implemented in this study for PDSS, blockchain gateway, and smart contract management.

Another approach addressed the authentication and decentralization of the IoT device gateway by implementing a basic interface using blockchain technology [112]. In addition, this architecture supports IoT infrastructure with lacking versatility and anonymity within its design. In addition to the interface, IP mapping for network nodes is included. The design environment consists of a customized hub, wired connections, and distributed ledger, preventing direct communication with the internet (only through the home server) and allowing the server to run on any device using a programming language (Node.js). The home server conducts the process of obtaining data (collection) and monitoring devices. The proposed design consists of four parts: smart device, home router, home server, and remote service. The process starts when data are generated from a smart device and passed to the home router for port forwarding. The data are transmitted to the home server; in this step, the data are parsed, and the request is appropriately encrypted. Unused data by remote services are removed, and the home router receives the request. It allows data to be sent to the remote service, and the remote service parses the incoming data and decides the proper action. The home router receives the data using port forwarding from the allowed service and transfers the action to the smart device to be performed. Further considerations can be made to improve the security by providing a flexible interface from the manufacturers, and a list of IP addresses must be included to identify legitimate access requests. Further experiments can be conducted to determine how robust this design is against possible IoT infrastructure attacks.

### 4.5. IoT Information Systems

Data integrity is one of the main areas researchers focus on when designing security and privacy models. A novel privacy-preserving model is proposed to address this area in IoT information systems using blockchain, bilinear pairing, and a Lifted EC-ElGamal cryptosystem [113]. This work is completed in a cloud environment for outsourced data integrity. To support the aim of this study, they proposed a protocol that achieves correctness, privacy, security, dynamic updating, and public verification. This scheme includes a data-checking model that consists of a Data Owner (DO), Key Generate Center (KGC), cloud server, and auditors. Any part can act as the auditor for data integrity checking and receives a reward, but it has to own enough capabilities and expertise to perform this task. The outsourced data are represented by the high volume of data that the DO has that forces the DO to request cloud storage. A trust model is also used to check the integrity of outsourced data. This model assumes that the DO, cloud servers, and auditors are semi-honest, making it suitable for practical application. Compared to other approaches, this scheme supports dynamic auditing; it performs remote data integrity checks without needing a third-party auditor, eliminating data privacy leakage.

Another study presented a framework for a sharing security mechanism for IoT information systems by adapting and combining transaction blockchain and data blockchain (double-chain model) [114]. Using partial blind signature algorithms, privacy protection and transaction efficiency can be enhanced. A node cooperation technique based on the dynamic game method is proposed to prevent any local dominance of malicious behavior. To reach Bayesian equilibrium, the node’s institutional reputation value is reported to estimate the state of the unknown node; it is also used to identify malicious nodes and correct their overall report. The authors improved the PBFT algorithm to eliminate the issues found in the common consensus algorithms, such as requiring intensive resources and being computationally time consuming. In the total number of nodes that calculates consensus, f=(n−1)/3 must be the maximum number of error nodes. They considered reputation and computing power as one solution for accounting nodes or legal currency (digital currency) as the other. A coin center is set up using the cloud service to avoid multiple payment issues and privacy breaches. A distributed accounting system is added to the chain to allow the traceability of bills.

### 4.6. Management Systems

In any IoT ecosystem, it is crucial to compute the assets (operations, users, devices, etc.) and their provenance. Thus, a secured ID management system based on blockchain technology is proposed to tackle current security and privacy issues [131]. The authors developed a proof-of-concept prototype in a business case scenario. This prototype consists of three stages; the first stage discusses how the data can be managed and modeled to be stored securely on the blockchain. The second stage explains different rules developed using smart contracts for any agreement and monitoring these rules while the core transactions are also being processed. The third stage manages identity verification to address security and privacy aspects. Four types of smart contracts’ rules are used: computer ID management, software ID management, user ID management, and data backup ID management. The proposed prototype can be further explored for large-scale businesses in terms of adaptability and extendability.

SmartDID [132] is a distributed identity management system proposed due to a lack of a systematic proof system and issues in IoT, such as privacy, security, and resource limitations. The authors constructed a distributed identity system to support certain features, for example, supervisability, unlinkability, and Sybil attacks resistance. The authors considered the following: to hide privacy information, cryptographic credentials and plaintext are used to create a dual-credential model. In addition, a zero-knowledge scheme is used as a verification mechanism, and a commitment scheme is used for encryption to secure cryptographic credentials. However, there is a possibility of a Sybil attack, and such methods are unable to hide attribute linkage. Thus, a distributed system with multiple pseudonymous userIDs and one unique masterID is designed to address the mentioned issues. The system model is comprised of supervisors, verifiers, issuers, holders, and a consortium blockchain. IoT devices can act as verifiers to verify credentials and publish access policies, while the committee acts as a credential issuer to sign and issue credentials. SmartDID was evaluated and compared with two other approaches in terms of proof generation and credential generation times, and SmartDID achieved the best performance.

### 4.7. Other Works

#### 4.7.1. Cloud Environments

Protecting the data-sharing process in cloud environments is crucial to eliminate any leakage or breach. A secure data-sharing scheme that integrates blockchain technology, Information-Centric Networking (ICN), Identity-Based Encryption (IBE), and Proxy re-encryption (PRE) is proposed [128]. PRE allows transforming a file from delegator to delegatee by encrypting the file with the delegator’s public key. In IBE, the email (identity) is used as the public key for encryption. Confidentiality is achieved using a secure access control framework; security and privacy are ensured using a complete protocol based on the PRE scheme; network bandwidth utilization and enhanced data delivery are ensured through proxy nodes. The designed platform consists of data producers responsible for generating the data, cloud service providers (CSPs), data owners to whom the data belongs, and data users representing the recipients of the information. Performance analysis and comparison indicate that the proposed scheme is efficient compared to other works.

It is possible to convince the verifier that a particular assertion of information is correct without involving any valuable information; this is called zero-knowledge proof, which can be generated using the Zero-Knowledge Succinct Non-Interactive Argument of Knowledge (zk-SNARKs) tool. This approach utilizes zero-knowledge proof combined with smart contracts to allow trusted sharing between semi-trusted cloud servers, Cloud Service Providers (CSPs), and data owners [129]. Proxy re-encryption technology is integrated into this model to provide authorized CSPs with a secure data-sharing model. The proposed system model contains six entities: the blockchain network, smart contracts, private key generator (PKG), semi-trusted cloud server, cloud service organization, and data owner. Performance analysis is completed for content privacy, identity privacy, data validity, and traceability. This model is still in the research phase and has not been implemented yet.

#### 4.7.2. Fog Computing

Using alliance chain and fog computing, a blockchain-based distributed access control system is proposed to offer security for IoT networks using a combined LSB and MLNCML encryption scheme [125]. This system utilizes fine-grained and dynamic access control to eliminate a single point of failure. In the proposed system architecture, edge nodes connect smart devices to the internet and allow resource requestors to access the services in the smart devices. The alliance chain is formed using all blockchain nodes, including the edge nodes. Thus, edge nodes offer a distributed manner when performing access control decisions. In the proposed encryption scheme, the first encryption is performed by the chaotic series, and then the LSB algorithm obtains the encrypted image. Finally, secondary encryption is performed by the MLNCML. In the proposed access control model, ABAC, LSB, and chaotic encryption is used, and the authors included a detailed workflow of this model. Using alliance blockchain limits access to increase the security and safety of the system; it also ensures the validity of the data and prevents any tampering because the data are distributed on the alliance chain. From a security perspective, the authors discussed data privacy, data theft prevention, anti-data tampering, and file storage security. By comparing this work with other approaches, it offers dynamic access control as well as fewer start-ups and nodes; in addition, it does not require paying remuneration, the proportion of voting rights is not artificially needed, less computing power is required, and it features enhanced management. This system can be improved by including a lightweight consensus protocol, avoiding a single point of failure by including smart contracts for distributed access control and ensuring the security and credibility of edge nodes using trusted computing technology.

An IoT distributed middleware layer is integrated with permissioned blockchain to construct a lightweight and robust system for managing IoT data [126]. In the proposed system, the IoT management layer handles IoT data instead of assigning end devices or a central authority to handle this task. This system adapts a networked smart object (NOS) to manage heterogeneous sources’ data and evaluate it, since it is a flexible and cross-domain middleware approach. NOS adapts the MQTT protocol working mechanism (publish and subscribe) for information exchange. NOS can enforce access control rules using a sticky policy mechanism. In the investigated scenario, low power consumption, low latency, and a limited number of users are required; thus, a Byzantine Fault Tolerant (BFT) replication consensus algorithm is used since it fits such requirements. Evaluation experiments are completed to recognize the proposed system’s robustness regarding access control, confidentiality, integrity, and performance under malicious attacks.

#### 4.7.3. Edge Computing

A study addressed the access control issue in edge computing applications called TrustChain [90]. TrustChain aims to eliminate centralized processing delays and privacy problems while preserving IoT networks’ resources. TrustChain is based on the following characteristics: interoperability, compatibility with different models, enhanced scalability and privacy, reduced delay and operation with an efficient mining scheme. This model describes various services offered using this model, including trust services (validator management, trust management, auditing and accountability, and trust data repository), blockchain services (consensus management, distributed ledger, ledger storage, P2P protocol, cryptographic services, and IPFS storage), smart contract services (registry, secure container, and life cycle), membership services (registration, ledger identities, and resource identities), and policy services (privacy, access control, and consent management).

#### 4.7.4. Reputation Systems

To address the issues of a single point of compromise and failure, a decentralized trust model is proposed [127]. Using past interactions with public fog nodes, this approach allows for maintaining the reputation of network users. The architecture of this model consists of the following components. (1) The fog node has a unique Ethereum address that associates with a list of evaluations that contains information such as generic reputation score, cost of service, storage, and latency. (2) The IoT client provides feedback after every interaction with any fog node, and the credibility factor measures the fairness and honesty using several provided ratings, consistency, and trustworthiness. (3) Five types of smart contracts are used, namely custom reputation, credibility, reputation, node management, and client registration. The authors included a diagram to illustrate the workflow of computing the reputation of fog nodes. The process is discussed in detail between the blockchain platform, off-chain fog nodes, and front-end DApps. This approach is implemented using the Ethereum blockchain to ensure network security, immutability, and validation. Based on the evaluations for common security vulnerabilities, the results show that the smart contracts are safe against them, and there are no major security issues when running software that identifies security vulnerabilities.

#### 4.7.5. Mobile IoT Applications

A blockchain-based architecture is explicitly proposed for mobile IoT applications to enhance the security and privacy of users’ data by implementing user-controlled privacy [115]. This architecture consists of Storj, MQTT, and blockchain. The latter is used to exchange information and store metadata and non-sensitive information. MQTT is a lightweight communication protocol that serves as middleware between the blockchain core and IoT devices. Finally, sensitive data are stored in a storage system represented by Storj. To enhance the security of this model, two types of blockchains (each with its smart contracts) are used: public and private. Smart contracts and the proxy are used to transfer requests from public to private blockchain. Access Control Lists (ACLs) perform the access control process for authorized nodes. Specific nodes, referred to as bridging nodes, are participating in handling this process to allow intermediation and handle requests in both blockchains.

In terms of the cryptography scheme, this architecture utilizes the advantages of the Elliptic Curve Integrated Encryption Scheme (ECIES) framework with secp256k1 [138]. Many interactions and processes are discussed in detail in this study; for example, the sensor controller performs the process of sending ACL, exchanging keys is completed by the MQTT broker, registering ACL occurs within the PBVU smart contract, the proxy sets the destination node and encryption key, event creation is completed using smart contracts of a public blockchain, and finally, public nodes are responsible for parsing and decryption procedures. Many concepts have discussed how this model achieves authorization, authentication, data confidentiality, user-controlled privacy, user anonymity, and location and data privacy. Based on the evaluations, all components can offer enhanced performance and deliver high security and privacy. It is possible to enhance this architecture by eliminating the disclosure of ACL using a privacy-preserving mechanism, investigating the single point of failure, and high availability mechanisms. This approach can be implemented and evaluated in a cloud environment and with other implementations to explore how it affects network throughput. Finally, a mechanism can be integrated into this approach to allow adding new nodes to the system. This mechanism must include policies and rules to handle such operations.

★ Further considerations, including open issues (future works) and remarks, are highlighted in Table 8. We can note that 79.07% of the included studies discussed open issues for further consideration, while 20.93% did not discuss if any further work is required. Some studies are only noted with “this approach is not implemented and evaluated yet” because it was presented as a use case [90,93,99,101,116,117,124,129]. We used two marks to differentiate between prons and cons: ★ and ❖, respectively. One of the most important features for security is to keep the system robust against attacks [95,101,114,122,123] and to handle any misbehavior [120]. Requiring less computing power [125] is also essential, since using blockchain technology might result in high power consumption. Supporting parallel computing makes the proposed model more efficient [95]. In terms of scalability, one model could offer an enhanced scalability [90], while the other model caused a scalability issue [112]. The detailed description is illustrated in Table 8.

Additionally, the following remarks can be taken into consideration:

- It is important to realize a mechanism to handle exchanging messages among network nodes to reduce the overhead.
- Eliminate using a centralized server that conflicts with the decentralization feature which blockchain technology offers.
- When designing a BIoT scheme, the scalability and confidentiality of data must be proven to consider the approach efficient.
- In several studies, the blockchain side is neglected, and there is insufficient information about the type of blockchain, platform, and consensus algorithm.
- It is crucial to evaluate and validate the proposed approach regarding security and resiliency against different attacks.
- The computation cost and communication overhead must be deeply investigated and studied.
- Although some studies enhance network privacy and security, it affects network performance metrics negatively, such as throughput and latency.
- A standardized evaluation manner needs to be followed; a public blockchain platform can be used to compare the performance of different proposed approaches.

## 5. Lessons Learned

A wide range of sectors has benefited from the use of blockchain technology. IoT’s security and privacy concerns are still being explored when blockchain technology addresses these aspects. In addition, blockchain allows a variety of security and privacy-preserving models for the Internet of Things applications, offering decentralization, anonymity, autonomy, transparency, privacy, security, collective verification, and many more. Since IoT devices are constrained with low capabilities, they require further consideration when designing an approach to enhance their performance. The integration of blockchain technology and IoT has also introduced some limitations that must be considered: for example, scalability, storage capacity, resource utilization, the method to deploy smart contracts, and legal issues.

Blockchain and IoT factors are discussed in depth in this study through a systematic literature study. It also offers several existing solutions and blockchain applications for various IoT areas. Decentralization, auditing attributes, anonymity, and persistence are just a few of the advantages of blockchain technology that made academic research and industrial domains very attractive. This study has opened new doors for future research to address many important issues that need further investigation.

As shown in the above tables, blockchain technology targets many applications and sectors. However, a missing standard needs to be initialized when implementing such approaches because an IoT network requires many parameters to be considered in the pre-built or theoretical part design. For example, it is tough to decide what model is more efficient than others when there is no public platform on which these technologies can be integrated and built. Hence, it will require a new type of blockchain, for example, consortium blockchain, with extra features to handle IoT demands. In our opinion, consensus algorithms are one of the main limitations or drawbacks in such models because using generalized algorithms does not allow the system to operate at full capabilities (the performance level it was designed to operate at). In [117], the authors investigated the performance with different consensus algorithms, which offers an advantage in determining which algorithm results in higher security and performance.

In terms of the evaluations, we can note that there are still many distinct parameters that have been used; this means that it offers various measurements that can be used to evaluate the proposed models, and different programming languages can be used. However, there is still a huge gap between each study, making it tough to address specific issues because various tools might result in different values for each evaluation metric. Many studies still miss the evaluation environment; these approaches can be investigated in different environments and various metrics.

Many issues in the included research papers have been addressed in our study. For example, we can investigate how to exchange messages between devices and servers using a lightweight mechanism, eliminate a single point of failure, design a model to handle large-scale networks, assign the mining process to specific nodes to achieve an efficient mining scheme, propose a consensus algorithm that is specifically designed to handle blockchain and IoT integration, eliminate privacy concerns when implementing the model in a real-time environment, reduce resource requirements, improve data accuracy through noise reduction, and handle legal issues when implementing an approach in a domain that require high privacy such as the healthcare sector.

Finally, this study aims to provide an overview and further directions to researchers interested in the BIoT concept. In addition to the systematic literature, we offered a technical perspective on different studies included in this review. Based on the data collected, our findings demonstrate that from 2018 until 2022, researchers are primarily interested in designing approaches for the healthcare domain, followed by smart environments and generic approaches. Such aspects include universal and endless possibilities for researchers to improve and enhance current designs. From our perspective, future research seeks business and industry sides as it grants the researcher the ability to implement and validate the work in a real-time environment. In addition, healthcare and finances are critical in BIoT applications. EHRs can be managed remotely and securely while preserving patients’ privacy and creating a decentralized government that controls cryptocurrencies and the prediction marketplace. After all, many issues have not been addressed yet in which the combination of blockchain and IoT can offer the optimum solution; however, proper decisions must be taken into consideration for each target application, such as blockchain type, platform, consensus algorithm, power consumption preferences, and network latency requirements. Further details on possible research directions can be found in the discussion of Table 8.

## 6. Conclusions

IoT dramatically facilitates people’s daily lives by exchanging data and making complete decisions. However, it raises sensitive issues of security and privacy at the same time. Security and privacy concerns in the Internet of Things (IoT) could be efficiently addressed by blockchain technology. This paper conducts a systematic literature review of the state-of-the-art blockchain technology achievements that have been proposed to enhance IoT’s security and privacy aspects. In this review, we discussed the basic principles of technologies, including their architecture, protocols and consensus algorithms, characteristics, and the challenges of integrating them. We overviewed the methodology of our review, including the search strategy, eligibility criteria, and selection results. Our findings are presented in a systematic literature manner based on the characteristics of included papers. Our results (mainly focused on the targeted applications or domains) show that 27.9% of the included studies focused on healthcare domain, 18.6% focused on proposing generic approaches, and 23.25% aimed to target smart environments applications divided into 9.3% for smart home applications, 6.98% presented systems designed to target smart cities, and 2.32% for each of the following: smart factory, smart traveling, and smart agriculture. Furthermore, studies of the IoT device gateway, IoT information systems, management systems, cloud environment, and fog computing have been carried out (4.65% for each separate application). Finally, the rest (≈6.99%) aimed to address edge computing, mobile IoT applications, and reputation systems. We also showed various characteristics for each study, such as the main goal or objective, development level, a blockchain platform, blockchain type, consensus algorithm, evaluation environment and metrics (if found), notes for each study which contain prons and/or cons, and future works (open issues). All articles are also discussed from an architectural and operational perspective. Finally, we identified significant gaps and future considerations that can be taken into account when integrating blockchain technology in the IoT domain.

## Figures and Tables

**Table 1:** Primary Information of the Included Papers.

Ref #	Year	Country	Publication Type	Publisher
[90]	2019	UK	Journal	**Wiley–Hindawi** Wireless Communications and Mobile Computing
[91]	2020	India	Journal	**IEEE** Internet of Things
[92]	2019	China	Conference	**IEEE** Fourth International Conference on Data Science in Cyberspace
[93]	2018	USA	Conference	**Springer** International Conference on Computational Social Networks
[94]	2019	China	Journal	**Elsevier** Future Generation Computer Systems
[95]	2019	China	Conference	**Springer** International Conference on Smart Blockchain
[96]	2020	China	Journal	**IEEE** Wireless Communications
[97]	2022	China	Journal	**IEEE** Internet of Things
[98]	2022	Saudi Arabia	Journal	**MDPI** Electronics
[99]	2019	Poland	Journal	**MDPI** Sensors
[100]	2021	India	Journal	**IEEE** Internet of Things
[101]	2019	Iran	Conference	**IEEE** Canadian Conference of Electrical and Computer Engineering
[102]	2019	China	Journal	**IEEE** Internet of Things
[103]	2018	Egypt	Conference	**Elsevier** The 9th International Conference on Emerging Ubiquitous Systems and Pervasive Networks (EUSPN)
[104]	2019	China	Journal	**IEEE** Network
[105]	2022	China	Journal	**IEEE** Internet of Things
[106]	2022	Morocco	Journal	**IEEE** Transactions on Computational Social Systems
[107]	2022	Nigeria	Journal	**IEEE** Systems Journal
[108]	2022	Pakistan	Journal	**IEEE** Access
[109]	2022	South Korea	Journal	**MDPI** Sensors
[110]	2022	Algeria	Journal	**IEEE** Access
[111]	2018	Taiwan	Journal	**IEEE** Access
[112]	2021	Serbia	Journal	**Elsevier** Energy Reports
[113]	2020	China	Journal	**Elsevier** Information Processing and Management
[114]	2019	China	Journal	**Elsevier** Future Generation Computer Systems
[115]	2021	Portugal	Journal	**MDPI** Sensors
[116]	2018	Mexico	Conference	**IEEE** International Smart Cities Conference (ISC2)
[117]	2019	Cyprus	Book	**Elsevier** Smart Cities Cybersecurity and Privacy
[118]	2020	Australia	Journal	**Elsevier** Computers and Security
[119]	2019	China	Journal	**IEEE** Transactions on Industrial Informatics
[120]	2018	Vietnam	Conference	**IEEE** International Conference on Advanced Computing and Applications
[121]	2020	India	Journal	**Elsevier** Future Generation Computer Systems
[122]	2019	Australia	Journal	**Elsevier** Journal of Parallel and Distributed Computing
[123]	2021	Australia	Journal	**IEEE** Access
[124]	2018	China	Symposium	**IEEE** Symposium on Service-Oriented System Engineering (SOSE)
[125]	2021	China	Journal	**Springer** Cluster Computing
[126]	2022	Italy	Journal	**Wiley** Concurrency and Computation: Practice and Experience
[127]	2019	UAE	Journal	**IEEE** Access
[128]	2021	China	Journal	**IEEE** Systems Journal
[129]	2022	China	Journal	**Wiley / Hindawi** Wireless Communications and Mobile Computing
[130]	2022	USA	Journal	**MDPI** Future Internet
[131]	2022	Australia	Journal	**MDPI** Systems
[132]	2022	China	Journal	**IEEE** Internet of Things

**Table 2:** Objective and Technology Readiness Level.

Ref #	Objective	TRL
[90]	TrustChain is an innovative privacy-protecting blockchain-based network to overcome the issues associated with existing IoT networks and investigates how to eliminate privacy and delay concerns while preserving IoT network resources	2
[91]	A detailed analysis is provided, including enabling technology and IoT technology integration. In a smart IoT system, a case study is implemented using an Ethereum-based blockchain technology	5
[92]	In this study, the authors presented a blockchain-based decentralized IoT system. They developed various access strategies and implement them using smart contracts	5
[93]	In order to ensure that users have full control over their data and can track how it is used by third-party services, a system model is developed. They also proposed a blockchain-based firmware update approach that helps prevent IoT device tampering from causing fraudulent data	2
[94]	Employing private information retrieval to present a Privacy-preserving Thin-client Authentication Scheme (PTAS) for IoT devices	5
[95]	Propose an efficient and secure authentication private protection scheme using blockchain technology and the AES algorithm to encrypt the original IoT information; this study provides an IoT information security protection strategy that may effectively address IoT data storage issues	5
[96]	Deal with the heterogeneity and scalability of IoT networks by proposing a three-dimensional architecture with unique data structures. In addition, they propose the 3D-GHOST consensus mechanism for spacechain	5
[97]	Propose a trust evaluation mechanism, PoT consensus algorithm, and privacy protection mechanism based on the commitment scheme and rign signature	5
[98]	This paper addresses the scalability, privacy, and security issues by introducing a multi-layer-blockchain-based solution. It eliminates the need of Trusting Third Parties (TTP) through multiple-chaincode-based access control	5
[99]	Propose a novel patient-centered electronic medical record access control framework based on modified blockchain models that take into account the IoT’s resource constraints	2
[100]	Propose a dual-layer blockchain-IoT privacy-preserving approach based on swarm exchange techniques to support the seamless and secure transmission of user data via secure swarm nodes of peer-to-peer communications	5
[101]	Propose a blockchain-based architecture for e-health applications that provides an efficient privacy-preserving access control mechanism	2
[102]	Healthchain is proposed which is a privacy-preserving scheme designed for the healthcare domain, this approach is large-scale and is used to conduct fine-grained access control for health data. Furthermore, introduce a distributed file system called InterPlanetary File System (IPFS)	5
[103]	Proposing a novel protocol named Pseudonym-Based Encryption with Different Authorities (PBE-DA), this paper aims to achieve perfect privacy-preserving EHRs in a multi-tier blockchain framework	5
[104]	This paper presents a privacy-protected blockchain-based system for medical image retrieval using layered architecture, a threat model, and a customized transaction structure	5
[105]	This work offers an architecture designed for IoMT and other possible applications. In addition, authentication schemes are introduced based on ECC and PUF to ensure the system’s privacy	5
[106]	A system that secures IoT devices in the healthcare domain using IPFS and blockchain technology. This system is designed to allow continuous monitoring for patients with chronic diseases	5
[107]	GaliMediChain is a healthcare system based on blockchain technology and garlic routing to enable secure sharing of health data (COVID-19). Furthermore, a new consensus mechanism (PoEoI) is proposed for blocks generation and miner selection processes	5
[108]	A medical Hyperledger Fabric-enabled blockchain-based architecture called BIoMT is proposed to increase the medical environemnt’s reliability while reducing the consumption of networking resources. Moreover, a customized consensus algorithm is designed to increase security and privacy	5
[109]	An implementation for critical systems to enhance privacy and security through combining Information Hiding Techniques (IHT), IoT, and blockchain technology	5
[110]	DSMAC is a decentralized system proposed to preserve the security and privacy of sharing medical records using Verifiable Credentials (VC), Decentralized Identifiers (DIDs), Attribute-Based Access Control (ABAC), Role-Based Access Control (RBAC), Self-Sovereign Identity (SSI), and blockchain	5
[111]	This approach aims to develop a blockchain-connected gateway for IoT devices that can adapt and securely retain the privacy preferences of users; security and privacy preferences can both be ensured by the proposed digital signature mechanism (PDSS)	5
[112]	This article discusses how the security gateway architecture of an IoT device can be provided with a basic interface using blockchain to allow decentralization and authentication. This offers IoT infrastructure with the required anonymity and versatility	5
[113]	By leveraging blockchain, bilinear pairing, and a lifted EC-ElGamal cryptosystem, this paper proposes a novel remote data integrity check approach for IoT information management systems that preserves privacy without requiring trusted third parties	5
[114]	This study presents a lightweight IoT data-sharing security framework based on a double-chain paradigm that combines data and transaction blockchains. To prevent malicious local dominance behavior, a dynamic game method of node cooperation is presented. This approach aims to improve privacy protection, data registration efficiency, and the PBFT consensus algorithm	5
[115]	The primary goal is to improve the privacy of users and their data by implementing user-controlled privacy using the anonymization characteristics of blockchain. This paper proposed an architecture to address privacy and security issues of IoT applications	5
[116]	A blockchain-based control access system integrated with IoT to improve smart cities services and performance	2
[117]	This research offered a smart city hybrid model that included five core features that the authors consider necessary to provide security and privacy	2
[118]	PrivySharing: a framework for secure, private IoT data sharing in smart cities via blockchain technology. By separating the blockchain network over multiple channels, data privacy is ensured	5
[119]	Transform the standard IoT architecture by introducing multi-center security and privacy blockchain architecture. Design the architecture’s data interaction and algorithmic processes. The specific solution is discussed using an automated manufacturing platform	7
[120]	A smart home-based IoT-Blockchain (SHIB) approach is proposed to address privacy, security, and authentication challenges	5
[121]	ELIB is a smart home model that is based on an overlay network that validates dedicated security and privacy by merging resources with high levels of capability into a public BC. This approach consists of three levels: DTM scheme, CC model, and consensus algorithm	5
[122]	This paper proposes a lightweight scalable blockchain (LSB) approach that focuses on achieving decentralization and optimizing the performance for IoT requirements through overlay networks. A time-based consensus algorithm is also proposed to reduce the delay and mining processing overhead	5
[123]	In order to develop a robust framework for smart home systems, they present an authentication model that integrates attribute-based access control with smart contracts and edge computing. In addition, they designed a Stochastic Gradient Descent (SGD) algorithm	5
[124]	In response to the need for data mining and analytic activities in IoT, a privacy-protected and inter-cloud data fusion platform based on JointCloud blockchain is proposed in this paper	2
[125]	Blockchain, fog computing, and the alliance chain concept are used in this research to provide a distributed access control solution for IoT data security that relies on LSB (Least Significant Bit) and MLNCML (mixed linear and nonlinear spatiotemporal chaotic systems) techniques	5
[126]	This works presents a cross-domain access control scheme that provides reliability and security. The distributed access control is achieved using blockchain and NetwOrked Smart object (NOS) middleware	5
[127]	A decentralized trust model is proposed in this research in order to maintain the reputation of publicly available fog nodes. The public fog nodes’ reputation is preserved by the opinions of previous users and their interactions with them	5
[128]	Secure the data sharing in cloud environments using a proxy re-encryption approach by combining PRE with blockchain technology, information-centric networking, and identity-based encryption	5
[129]	This article proposed a privacy protection scheme based on a combination of smart contracts and zero-knowledge proof in order to provide an effective use of data while maintaining data privacy and validity. Furthermore, in order to share the data safely and offer consistency between owners and cloud service providers, a proxy re-encryption technology is enclosed	2
[130]	A smart agriculture prototype is designed using cloud and blockchain to provide remote monitoring and alert mechanisms to farmers in real time	5
[131]	In order to overcome certain security and privacy issues in IoT ID management systems, this paper proposes a proof-of-concept blockchain-based modeling prototype	5
[132]	A distributed identity management system called SmartDID is proposed to address the lack of a systematic proof system and resource limitations for IoT devices	5

**Table 3:** Generic approaches articles: blockchain characteristics, evaluation environment, and metrics.

Ref #	Blockchain Platform	Type of Blockchain	Consensus Algorithm /Protocol	Evaluation Environment	Performance Evaluation Metrics
[91]	Generic Ethereum (Extension)	Public or Private	PoBT	Solidity platform, Ethereum’ web3.js, different types of sensors connected to Raspberry Pi	N/A
[92]	EOS	Private	DPoS	EOS system, desktop computers as gateways, gateways complied contract with C++ language	Max, min, and average time for contract deploy and execution, transaction package and validation
[93]	Ethereum or Hyperledger	Private (Hyperledger)	✖	N/A	N/A
[94]	Certcoin	Public	PoW	On a mobile phone with specific hardware parameters, these operations are tested and programmed using JAVA	Functionality, Computational overhead of thin-client and full node users, Communication overhead
[95]	Ethereum	Consortium	IPFS used in data storage module	Performance test on AES with a specific hardware specifications desktop, private IPFS network for information storage, Lena image, smart contracts are deployed on Ropsten Testnet test network	AES encryption and decryption rate, Delay of node joining the network
[96]	Spacechain	Private	3D-GHOST Modified PoW	Python 3, simulator: Compiled automatic transaction generator, testbed: Multi-miner P2P network test consist of 50 cloud virtual machines	Defense effect of selfish mining and DDoS attack, Network performance, Network throughput (block creation rate and block size limitation), Scalability
[97]	Ethereum	Public	Proof of Trust (PoT)	Solidity platform, Ethereum virtual machine (EVM), Remix IDE, network with 20 nodes (four types of nodes)	Trust evaluation mechanism, Running time, Expected mining cost
[98]	Hyperledger Fabric	Permissioned (Consortium)	PBFT	Hyperledger Fabric (v1.4.4) as a blockchain platform, Docker engine (v19.03.8, build afacb8b7f0) for runtime, Docker-compose (1.25.0) for image configuration, Node (v10.24.0) to create clients, Golang language (go1.16.2) for smart contracts creation, Hyperledger Caliper [133]	Throughput, transaction latency

**Table 4:** Healthcare Articles: Blockchain Characteristics, Evaluation Environment, and Metrics.

Ref #	Blockchain Platform	Type of Blockchain	Consensus Algorithm /Protocol	Evaluation Environment	Performance Evaluation Metrics
[99]	Generic	✖	✖	N/A	N/A
[100]	Swarm	Private	IPFS	GnuPG, IPFS, Golang, Five types of IoT-based health sensor nodes	Time of loading, exchange, listening, announcement, and availability, IoT elements activity
[101]	✖	Private	PBFT	N/A	N/A
[102]	Userchain Docchain	Public (Userchain) Consortium (Docchain)	PoW (Userchain) PBFT (Docchain)	Simulate the user node with a smart phone, The experiment is built on the platform Android 7.1.1, Java is used for IoT transaction and key transaction, OS Windows 7 is used to measure doctor nodes and mining nodes Python is used for programming Docchain and Userchain	Effectiveness and feasibility (computation and communication costs for user transactions generation
[103]	Multi-tier platform	Public (it can be also considered a consortium, since it contains constrained and unconstrained nodes)	PBE-DA	MIRACL Library (security tools ) Linux Ubuntu 12.10 on a computer machine	Processing time from different sources and destinations
[104]	Ethereum	✖	✖	Geth is used as the Ethereum client	Transaction generation time with varying transaction capacities and image retrieval time
[105]	Hyperledger Fabric	Consortium	✖	Edge server (Ali cloud platform), SD or EU simulators (smartphone), Fabric platform with multiple nodes is used to evaluate the performance of a smart contract	Computation cost, Communication cost, Time cost of smart contract
[106]	Ethereum	Private (in experiments) Consortium (in problem formulation)	Proof of Authority (PoA)	Private Ethereum Clique Blockchain (PC) Private IPFS network (PC), Raspberry Pi 3 Model B, Smartphone (DApp interfaces), JSON-RPC protocol	Processing time for different operations
[107]	✖	Consortium	Proof-of-Epidemiology-of-Interest (PoEoI)	The implementation environment is available online	Total cost (time) to evaluate the system’s efficiency, adaptability, and robustness. Elapsed time to request or respond, total utility, probability (availability attack), consensus protocol evaluation: computation cost and number of nonces.
[108]	Hyperledger Fabric	Consortium	Customized lightweight PoW	Docker (Hyperledger Fabric), Applying a resource limit mechanism on network nodes, Hybrid network topology, Experiment over P2P network	Frequency evaluation of CPU usage, Computational fluctuations (cost), Ratio between number of medical transactions and the total number of connected devices, Rate of throughput, duty cycle, delay, and response
[109]	Hyperledger Fabric	Private	Proposed PBFT	Smart city network models using network simulator-3 (ns-3), Network topology using Python, GO-Ethereum	Network throughput using PBFT compared with the classical algorithm, Latency of fault peers, General latency of execution over the network
[110]	Hyperledger Indy [134] Hyperledger Aries [135]	Permissioned (Consortium)	Zero-Knowledge Proof (ZKP) [38]	Hyperledger Indy for identity management, Hyperledger Aries for digital credentials, Solidity and Hyperledger Ethereum to run smart contracts, ACA-Py as cloud agent, VON-network as a ledger browser, Docker community edition	Transaction time (DSMAC evaluation), Transaction throughput, Transaction latency, Cryptographic computations, Scalability, Sustainability

**Table 5:** Smart Environments Articles: Blockchain Characteristics, Evaluation Environment, and Metrics.

Ref #	Blockchain Platform	Type of Blockchain	Consensus Algorithm /Protocol	Evaluation Environment	Performance Evaluation Metrics
[120]	Ethereum	Private	PoW	Ganache, Remix, web3.js	Smart contract, Data privacy, Usage of tokens, Updating the policies, Misbehavior Judging
[121]	Simulated platform on NS3	Public	Time-dependent consensus algorithm	Cooja, Network Simulator 3 (NS3), C++ programming language	POW processing time, Time overhead, Energy consumption, Packet overhead
[122]	Simulated platform on NS3	Public	DTC	NS3, MinerGate	POW processing time, Request/response delay, Impact of the number of OBMs on security and packet overhead, Impact of PTV on the ability to detect appending attacks, DTM in the overlay
[123]	Ethereum	Private	PoW	Two sensors (temperature and LED), Python in google colab environment	Resource usage for single transaction, K-fold cross-validation, Accuracy
[116]	Generic	Public	✖	N/A	N/A
[117]	Ethereum	Public	PoW, PoS, PoA, and Proof of Vote (PoV) were investigated	N/A	N/A
[118]	Hyperledger Fabric	Private	SOLO and Kafka	Oauth 2.0, ClientApp, REST API, Hyperledger Composer-Playground, Hyperledger Caliper	Validation of ACL rules, Performance efficiency, Average commit time, Average throughput, Average latency
[119]	✖	Private	PoW	Four industrial robots, 3B Raspberry Pis (two sensing layers), Intel I5 platform (management hubs)	Real-time performance testing
[124]	JointCloud	Private	✖	N/A	N/A
[130]	Ethereum	Public (on Rinkeby Etherscan)	PoW	Arduino Sensor Kit, ESP32, AWS cloud, Ethereum Rinkeby Test Network	Latency of: Device-to-Cloud, Cloud-to-Blockchain, Blockchain-to-Client-Console, Alert Total

**Table 6:** IoT Device Gateway, IoT Information Systems, and Management Systems Articles: Blockchain Characteristics, Evaluation Environment, and Metrics.

Ref #	Blockchain Platform	Type of Blockchain	Consensus Algorithm/Protocol	Evaluation Environment	Performance Evaluation Metrics
				**IoT Device Gateway**	
[111]	Ethereum	Private	Ethereum-like	PDSS: Raspberry PI III (Debian 8), Java 8 for ARM, Eclipse 3.8, BC gateway: Desktop (Ethereum network), NVIDIA Shield TV as gateway, LG Nexus 5X as client application, Smart contract management: Asus ZenBook, JDK 8u151, Java EE 7	Computation cost for PDSS, Practical potential for BC gateway, Average time for smart contract management
[112]	✖	✖	✖	Node.js	AES, DES, and Triple DES are used to evaluate memory usage
				**IoT Information Systems**	
[113]	Generic	✖	✖	Python 3.7.1, Key size = 32 bits, ECC encryption using ElGamal algorithm	Probability of the illegality behavior detection, Average time of key generation compared to the size of key, Average time of six other elements
[114]	✖	Combination of public, alliance, and private chains	Improved PBFT	CentOS 7, JDK version is 1.80, Threshold signature (THS)	Throughput, Latency, Determination time, Transactions per second (TPS), Node density, Routing protocol performance in blockchain IoT low-speed environment
				**Management Systems**	
[131]	✖	Federated (Consortium)	✖	Solidity, Kaleido platform	Transaction monitoring, CPU time and utilization
[132]	FISCO BCOS	Consortium	PBFT	Java 1.8, Fisco Bcos platform, 28.5 Mbps /11.21 Mbps bandwidth, 10 ms average communication delay	Credentials generation, Proof generation, Proof time, Time per type of credential, Time for range credential, Block generation time, Credentials size, Average verification time, Time for credentials verification

**Table 7:** Other Articles: Blockchain Characteristics, Evaluation Environment, and Metrics.

Ref #	Blockchain Platform	Type of Blockchain	Consensus Algorithm/Protocol	Evaluation Environment	Performance Evaluation Metrics
				**Cloud Environments**	
[128]	Hyperledger Fabric	Consortium	PBFT	jPBC library [136] for pairing, A super-singular curve, elliptic curve cryptography to implement group-based schemes, NIST P-256, web3.js to generate transactions	Computation cost, Encrypted data confidentiality, Data encryption computation time, Transaction latency
[129]	✖	✖	Combined RBFT and Raft	N/A	N/A
				**Fog Computing**	
[125]	Hyperledger Fabric	Private	✖	Go language, JetBrains developer tools, MATLAB, Lena picture	Bit error rate with chaos coding parameter, Bit error rate with encrypted image pixels
[126]	✖	Permissioned (private or federated/consortium)	BFT replication	NOS architecture, 14 data sources, Data rate between 10 and 20 packets per second, Rate change policy, Block dimension and generation time, Raspberry Pi platforms, MQTT broker, Smart home testbed	Storage overhead, Computing effort (CPU load), Latency
				**Edge Computing**	
[90]	TrustChain	Private	Proof of Trust (PoT), Trust+BFT	N/A	Object trust model, Data trust model, Privacy trust model, REK: Reputation, Experience, and Knowledge
				**Reputation Systems**	
[127]	Ethereum	Public	PoW	MythX, Sercurify analyzer, SmartCheck, Oyente, Remix IDE using solidity	Cost (of transaction), Performance analysis, Security analysis
				**Mobile IoT Applications**	
[115]	Ethereum	Private and Public	Private blockchain: PoA, Public blockchain: PoW	Python’s time library, NetHogs (version 0.8.6), psutil (version 5.8.0) python library	Time overhead, Bandwidth consumption, CPU and memory usage

**Table 8:** Future Considerations.

Ref #	Future Works (Open Issues)	Notes
[90]	✗	★ Efficient mining scheme ★ Significantly small mining delay compared to PoW ★ Enhanced scalability ★ Compatibility with IoT business models ★ Interoperability among several TrustChains ❖ Network overloading due to excessive exchange of messages between devices and server ❖ The centralized server is used for storage ❖ This approach is not implemented and evaluated yet
[91]	✗	❖ It does not support dynamic access control
[92]	✓	★ It supports the fast and secure insert of the device in the perception layer
[93]	✓	★ Improved access tracking ★ Provided efficient access control and data transparency ❖ This approach is not implemented and evaluated yet
[94]	✗	❖ (m-1)-private PTAS sacrifices little efficiency in exchange for safety improvement
[95]	✓	★ Simple ★ Support parallel computing ★ Error not passing ★ Not easy to attack
[96]	✓	❖ In order to achieve anonymity, public keys alone are not enough ❖ Zero-Knowledge Proofs
[97]	✗	★ Robust against several types of attacks ★ Low expected computation cost ★ Moderate scaling ★ Transparency and verifiability ★ Good resiliency ❖ Does not include trust and reputation management
[98]	✓	★ The proposed system includes auto-policy enforcement, on-chain policy management ★ It provides security, fee-less, trustworthy (without TTP), and scalability ❖ In order to eliminate network congestion and reduce the latency, machine learning algorithms can be integrated
[99]	✓	★ Increased security due to the hybrid apporach that combined many lightweight cryptographic primitives with public and private keys ❖ This approach is not implemented and evaluated yet
[100]	✓	★ Protection of EHR against fraud ★ Interoperability of EHR data formats ★ Simplification of current paradigms ★ Low cost ★ IoT data aggregator and sensor heterogeneity ❖ Not feasible for a large-scale network ❖ Block is mined instantaneously by the virtual nodes itself; thus, miners are needed
[101]	✓	★ High resiliency against public blockchain modification and DoS, modification, appending, and 51% attacks ❖ This approach is not implemented and evaluated yet
[102]	✗	★ This approach offers on-demand rescission, accountability, and improved privacy
[103]	✓	★ Using file and data sharing, this approach improved intersectoral collaboration ❖ In terms of development and administration, it requires further accountability
[104]	✓	★ Low latency ★ High feasibility ★ Enhanced image size ❖ Privacy concerns are still an issue when implementing in real-time environment
[105]	✓	★ The proposed architecture is not limited to healthcare domain only ★ Efficient and pairing-free authentication scheme ★ Guaranteed user anonymity with satisfied security requirements ❖ Certain security properties and efficiency metrics can be improved
[106]	✓	★ The proposed system offers confidentiality, integrity, privacy, and access control ❖ The authors suggests adding a fog layer between different system entities in order to process and filter the data
[107]	✓	★ New consensus protocol is introduced while maintaining the system’s robustness, efficiency, and adaptability ★ The proposed consensus protocol requires less computational cost than PoW and PoA ❖ Overall computation cost is not considered when this approach is implemented
[108]	✓	★ Reduces computational cost ★ Enhanced node transactions performance ★ This design offers provenance, transparency, security, and integrity
[109]	✓	★ This work promises higher security levels and lower execution time ★ This approach achieves consistency, security, availability, integrity, and transparency ❖ Tested for medical supply chain-based scenario only ❖ No description of encryption and decryption of OTH
[110]	✓	★ As compared to other models, DSMAC overcomes the others as it provides scalability, sustainability, data privacy, and emergency case ★ DSMAC includes access control methods such as identification, authentication, and authorization
[111]	✓	★ Using such an access control approach provides a non-repudiation feature and allows users’ preferences and device policies to be preserved without tampering.
[112]	✓	★ The authors included the advantages and several important remarks that can be added to the proposed approach to improve its performance ★ Flexibility to use all encryption algorithms ★ Intrusion prevention ★ Adding a new layer of security ❖ This approach will be considered useless if the database is corrupted in any form ❖ Scalability issue: the processing performance decreases as the number of smart devices increases
[113]	✓	★ Supports dynamic auditing ★ Satisfies the public verification and correctness ★ The used storage method offers many advantages such as reducing cost
[114]	✓	★ Anti-attack capability (10 types of attacks) ❖ When this system is implemented in certain industries, its performance must be improved, as well as the risk of privacy leaks must be addressed.
[115]	✓	❖ Single points of failure (due to using smart contract proxy and MQTT) ❖ Reduced throughput ❖ Increased latency by the evaluation scenario
[116]	✓	❖ This approach is not implemented and evaluated yet
[117]	✓	❖ This approach is not implemented and evaluated yet
[118]	✓	★ This approach offers better scaling than a single Ch blockchain system ❖ Massive resource requirement ❖ IoT device integrity mechanism is required
[119]	✓	★ Enhanced scalability and flexibility ❖ It might introduce large communication overhead
[120]	✗	★ High extension ability ★ Ability to handle misbehavior
[121]	✓	★ Reduced processing time ★ Low energy consumption ❖ Requires extra cost due to cloud usage ❖ Low scalability
[122]	✓	★ Decreases processing time and bandwidth compared to traditional blockchains ❖ Using DTC in small networks can make the network vulnerable to Sybil attack
[123]	✓	★ Resilient against modification, linkage attacks, data mining, and DoS attacks ❖ Extra added noise (trade-off between accuracy and privacy) which might result in reduced data accuracy
[124]	✗	❖ This approach is not implemented and evaluated yet
[125]	✓	★ The proposed approach requires fewer start-up and running nodes ★ Less computing power is required ★ It does not need to pay remuneration ★ Offers effective management of the rights
[126]	✓	★ This approach supports confidentiality, integrity, and resistance to attacks ❖ The proposed approach needs further testing with the common blockchain platforms such as Ethereum or Hyperledger Fabric with more complex environments in order to compare its performance with other approaches
[127]	✗	❖ Further evaluations can be performed using throughput and power consumption
[128]	✗	★ It offers confidentiality, decentralization, auditability, and low overhead for data owners ❖ High proxy overhead ❖ Does not include mutliple proxies ❖ Splitting re-encryption key scheme can be included
[129]	✓	★ Offers content and identity privacy, data validity, verfiability, and traceability ❖ The proposed scheme relies on trusted third paties ❖ This approach is not implemented and evaluated yet
[130]	✓	★ The proposed approach offers mininal network latency ❖ IoT gateway is not implemented ❖ This approach can work with Ethereum and PoW only
[131]	✓	★ Authentication and secure identity are provided ★ This prototype is cost-effective ❖ Scalability issue, this prototype is not designed to handle large scale operation, adaptability and extendibility can be further investigated
[132]	✓	★ The proposed system supports Credential Nested Verification ❖ PBFT caused communication overhead between nodes which leads to limited reliability due to increased latency and bottlenecks in network transmission

## Data Availability

Not applicable.

## Abbreviations

The following abbreviations are used in this manuscript:

IoT	Internet of Things
IP	Internet Protocol
TCP	Transmission Control Protocol
UDP	User Datagram Protocol
P2P	Peer-to-Peer
SPI	Serial Peripheral Interface
IGMP	Internet Group Management Protocol
ICMP	Internet Control Message Protocol
REST	Representational State Transfer
DDP	Distributed Data Protocol
OSPF	Open Shortest Path First
IGP	Interior Gateway Protocol
AS	Autonomous System
WSN	Wireless Sensor Network
RFID	Radio Frequency Identification
PoW	Proof of Work
PoS	Proof of Stake
DPoS	Delegated Proof of Stake
TaPoS	Transactions as Proof of Stake
BFT	Byzantine Fault Tolerance
PBFT	Practical Byzantine Fault Tolerance
DBFT	Delegated BFT
PoA	Proof of Authority
PoSV	Proof-of-Stake-Velocity
PoP	Proof-of-Personhood
PoB	Proof of Bandwidth
PoET	Proof of Elapsed Time
SCP	Stellar Consensus Protocol
PKC	Public Key Cryptography
PKC	Public Key Cryptography
THS	Threshold signature
TPS	Transactions Per Second
PoV	Proof of Vote
PoT	Proof-of-Trust
LSB	Lightweight Scalable Blockchain
ELIB	Efficient Lightweight integrated Blockchain
SGD	Stochastic Gradient Descent
LSB	Least Significant Bit
IPFS	InterPlanetary File System
DTC	Distributed Time-based
ECC	Elliptic Curve Cryptography
PDA	Personal Digital Assistance
IHM	IoT Health Manager
DAG	Directed Acyclic Graph
CV	Cardinal Value
DV	Data Validity
CD	Contact Degree
ACC	Access Control Contract
JC	Judge Contract
DTM	Distributed Throughput Management
CH	Cluster Head
OBMs	Overlay Block Managers
DAC	Discretionary Access Control Models
MAC	Mandatory Access Control Model
MSP	Membership Service Provider
JCC	JointCloud Computing
JCCE	JointCloud Collaboration Environment
DO	Data Owner
KGC	Key Generate Center
ACL	Access Control List
ECIES	Elliptic Curve Integrated Encryption Scheme
MLNCML	Mixed Linear and Nonlinear Spatiotemporal Chaotic Systems
PEP	Policy Enforcement Point
CCBCM	Consortium Blockchain Manager
AEBCM	Aggregated Edge Blockchain Manager
EBCMs	Edge Blockchain Managers
PoEoI	Proof-of-Epidemiology-of-Interest
IHT	Information Hiding Techniques
SSI	Self-Sovereign Identity
VC	Verifiable Credential
DID	Decentralized ID
ABAC	Attribute-based Access Control
RBAC	Role-based Access Control
DSMAC	Decentralized Self-Management of data Access Control
ICN	Information-Centric Networking
IBE	Identity-Based Encryption
PRE	Proxy Re-encryption
ZK-SNARKs	Zero-Knowledge Succinct Non-Interactive Argument of Knowledge
